# Supplementary figures and images for: Ventricular, atrial, and outflow tract heart progenitors arise from spatially and molecularly distinct regions of the primitive streak
Source: PLoS Biol. 2021 May 17;19(5):e3001200. doi: 10.1371/journal.pbio.3001200 (PMC8158918; doi:10.1371/journal.pbio.3001200)

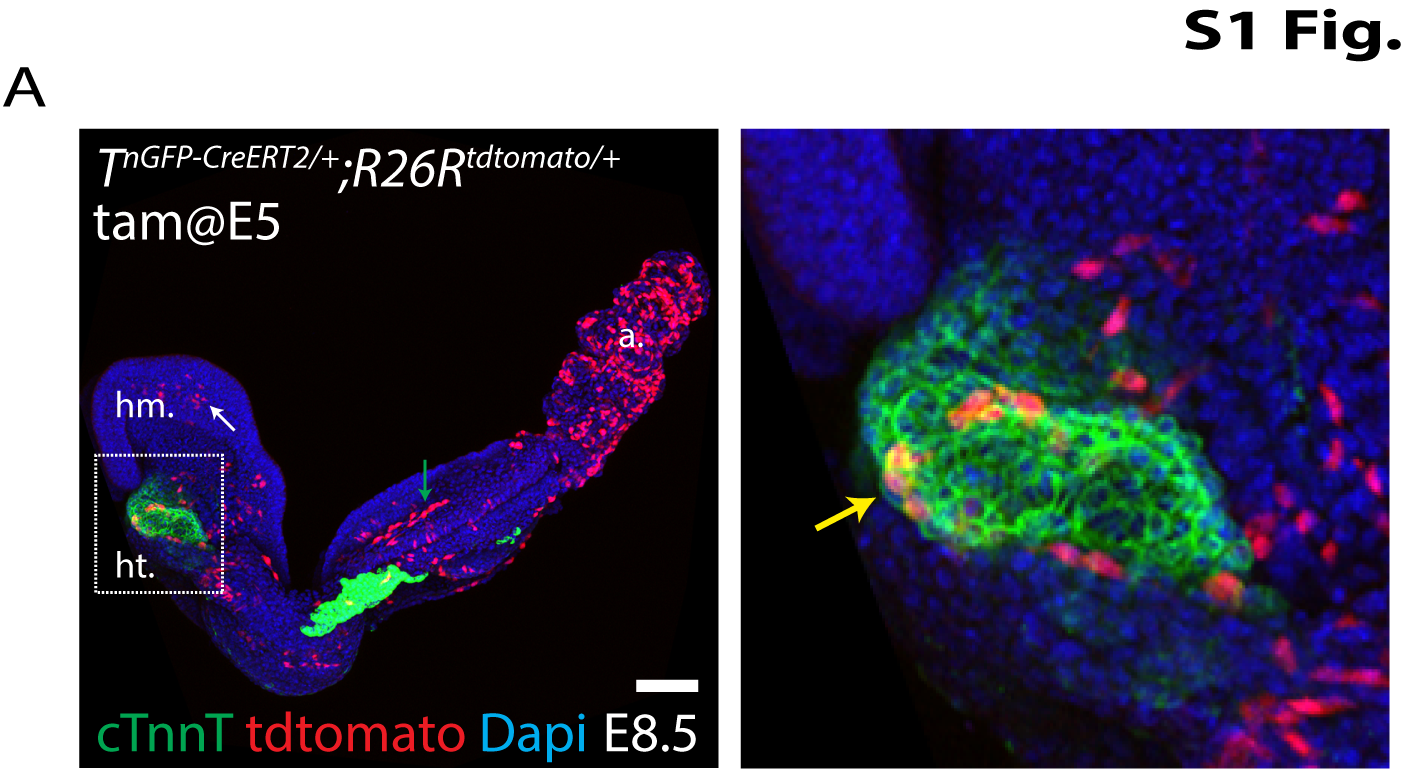

Supplement: S1 Fig — (A) The administration of a high dose of tamoxifen (0.08 mg/bw by oral gavage) at E5 in TnGPF-CreERT2/+; R26RtdTomato/+ mice leads to the presence of tdTomato-positive cells in mesoderm derivatives including cardiomyocytes (see yellow arrow in inset), head mesenchyme (red arrow), endothelium (green arrow), and allantois. a, allantois; hm, head mesoderm; ht, heart tube. Mouse were mated for a 2-hour period. Scale bar: 100 μm. (TIF) [file pbio.3001200.s001.tif]

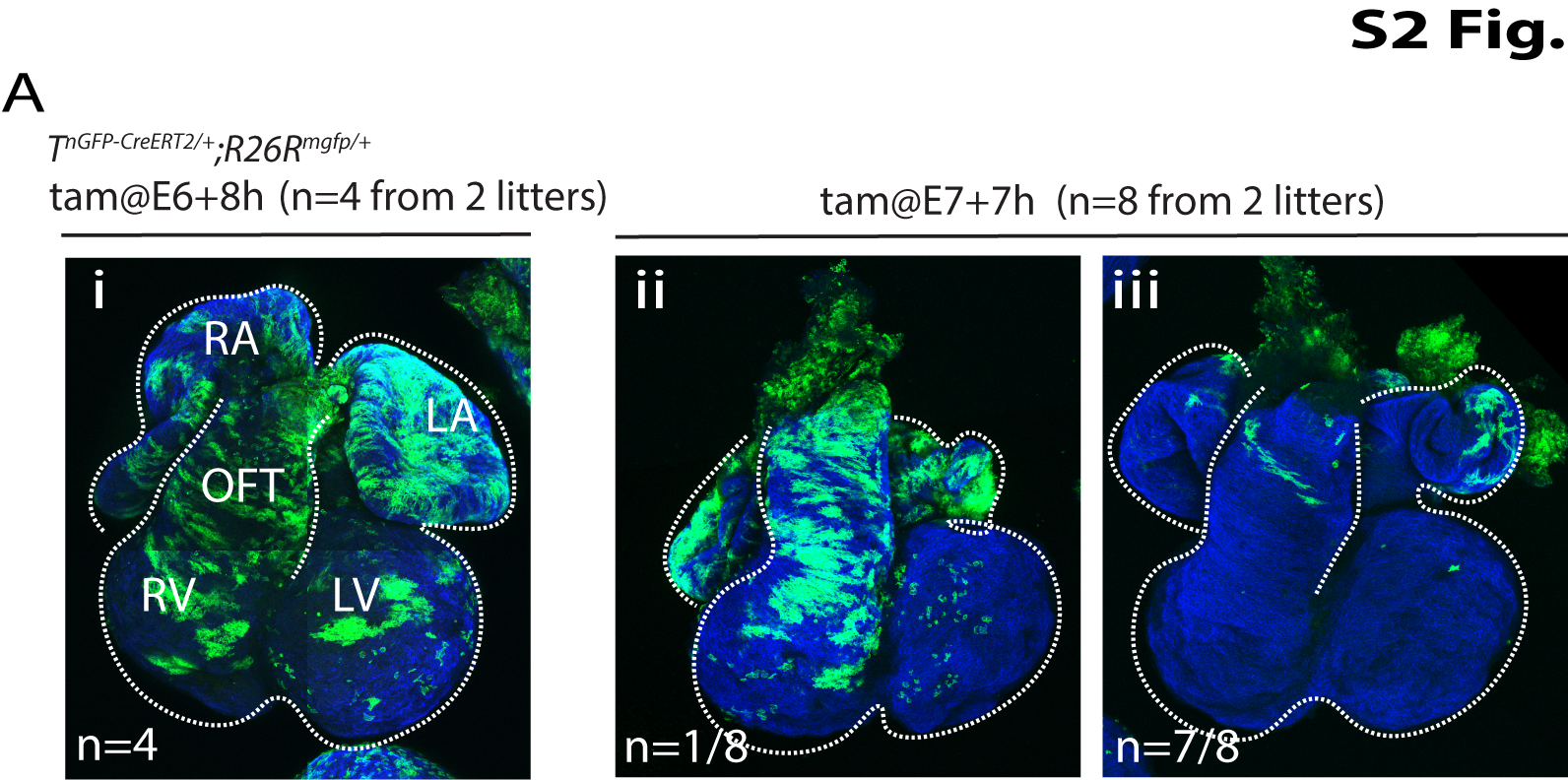

Supplement: S2 Fig — (A) Representative hearts resulting from the administration of tamoxifen at E6+8h (i) and E7+7h (ii, iii) in TnGPF-CreERT2/+; R26Rmtmg/+ immunostained with cTnnT to reveal the cardiomyocytes (blue). (TIF) [file pbio.3001200.s002.tif]

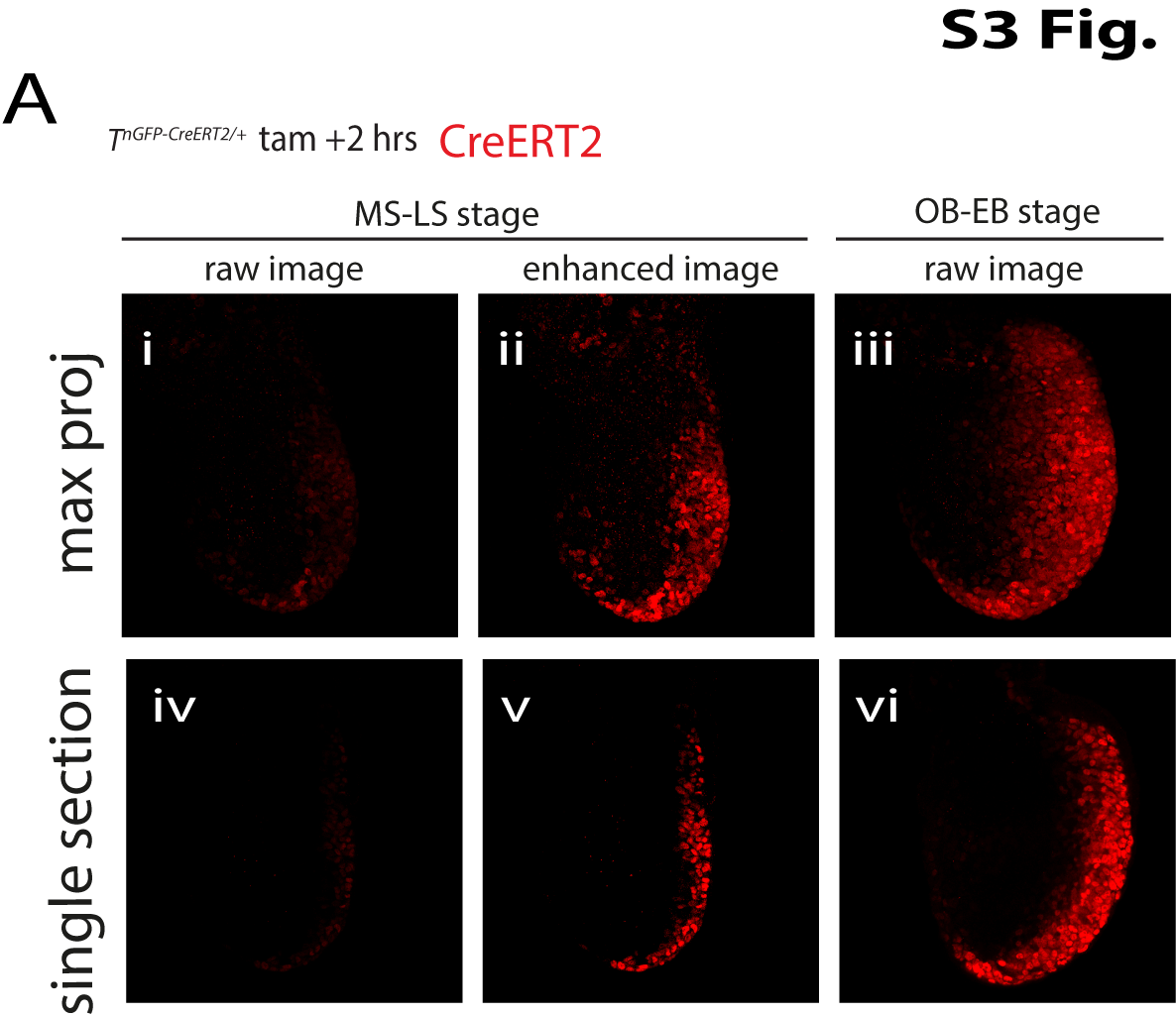

Supplement: S3 Fig — (A) Representative embryos resulting from a 2-hour pulse of tamoxifen via oral gavage (0.08 mg/bw) immunostained with oestrogen receptor. Embryos have been immunostained simultaneously and image under the same conditions. Maximum z-projection (i–iii) and single optical sections (iv–vi) are shown. (TIF) [file pbio.3001200.s003.tif]

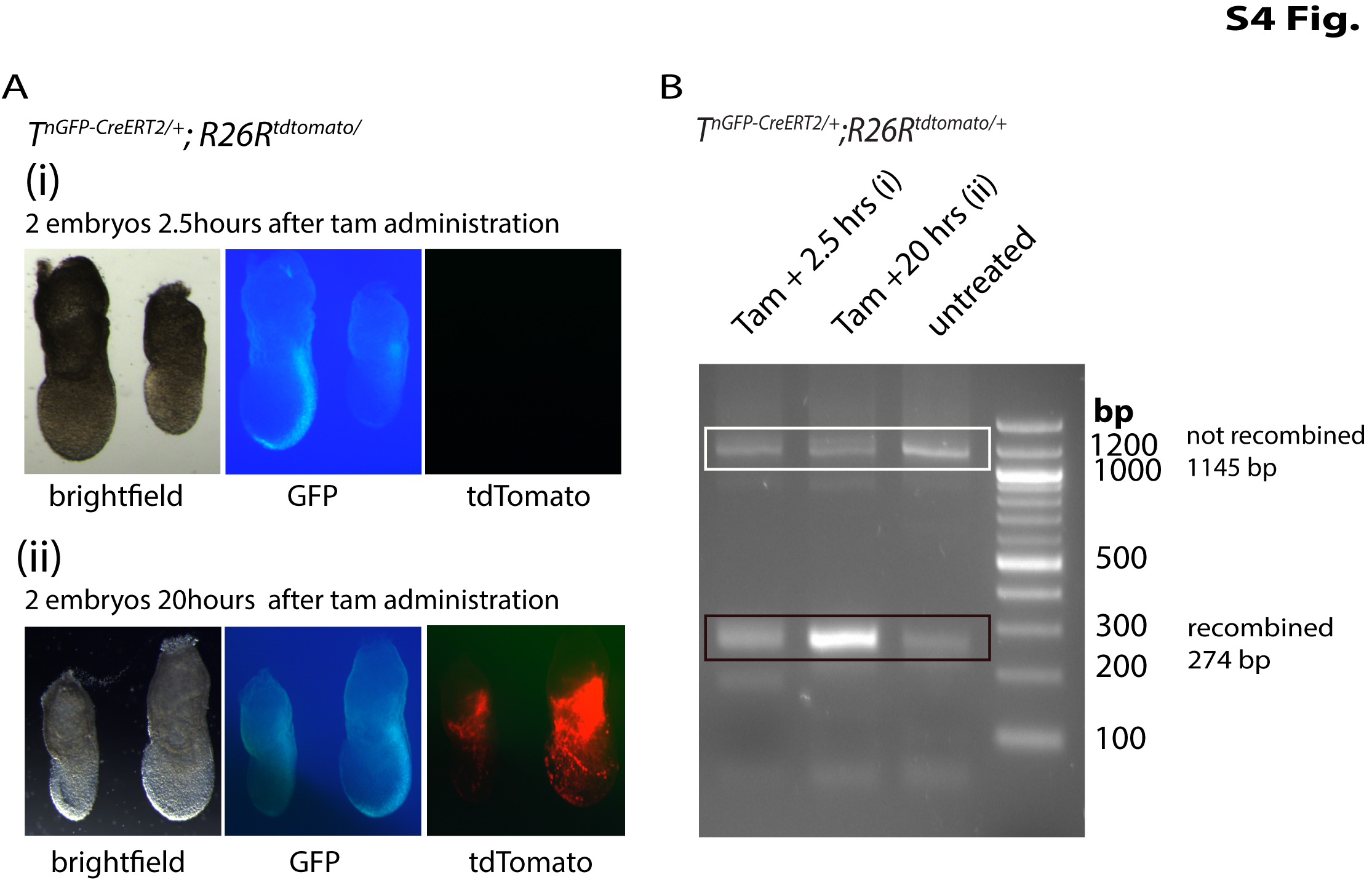

Supplement: S4 Fig — (A, B) PCR amplicons generated from the genomic region in which Cre-mediated recombination occurs from TnGPF-CreERT2/+; R26RtdTomato/tdTomato s embryos (A), resolved on an agarose gel (B). Before recombination, the PCR product is 1,145 bp (white rectangle); after recombination, it is 274 bp (black rectangle). Template gDNA was extracted from either an ear clip of an adult TnGPF-CreERT2/+; R26RtdTomato/tdTomato mouse (untreated) or TnGPF-CreERT2/+; R26RtdTomato/tdTomato embryos (i, ii) following oral gavage with Tamoxifen, as labelled. An increase in the proportion of the recombined band can be seen over time following Tamoxifen administration. The data can be found in S2 Raw image. (TIF) [file pbio.3001200.s004.tif]

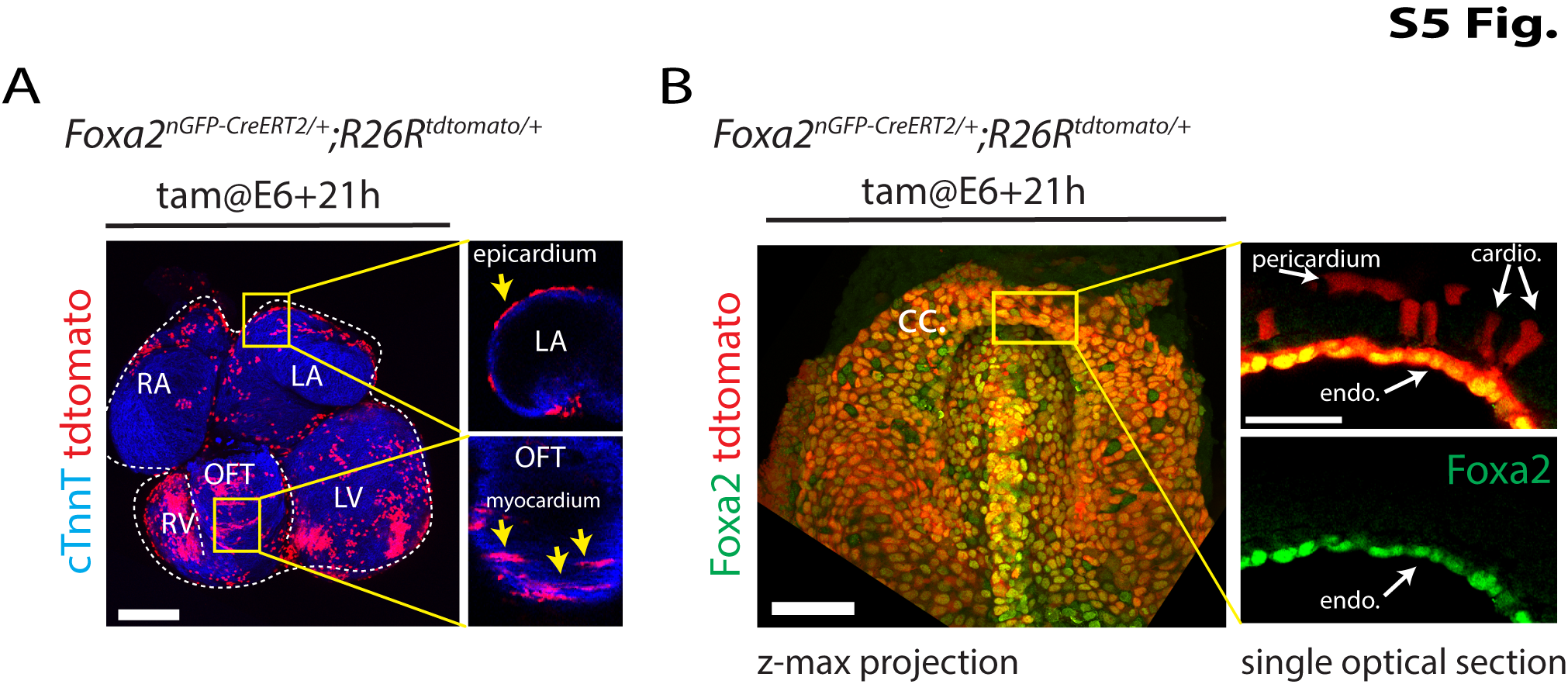

Supplement: S5 Fig — (A) Heart resulting from the administration of tamoxifen at E6+21h. View is ventral. tdTomato-positive cardiomyocytes are absent from the myocardium in the atria; however, contribution to the epicardium (yellow arrow) and myocardium (yellow arrows) in the ventricle and outflow tract is visible. (B) E8 embryo resulting from the administration of tamoxifen at E6+21h in Foxa2nGPF-CreERT2/+; R26RtdTomato/+ mouse and immunostained for Foxa2 (green). tdTomato-positive cells are localised in the pericardium, cardiomyocytes and endoderm but not in the endocardium. cardio, cardiomyocyte; CC, cardiac crescent; cTnnT, cardiac troponinin T; endo, endoderm; LA, left atria; LV, left ventricle; OFT, outflow tract; RA, right atria; RV, right ventricle. Scale bars: 200 μm in (A) and 100 μm in (B). (TIF) [file pbio.3001200.s005.tif]

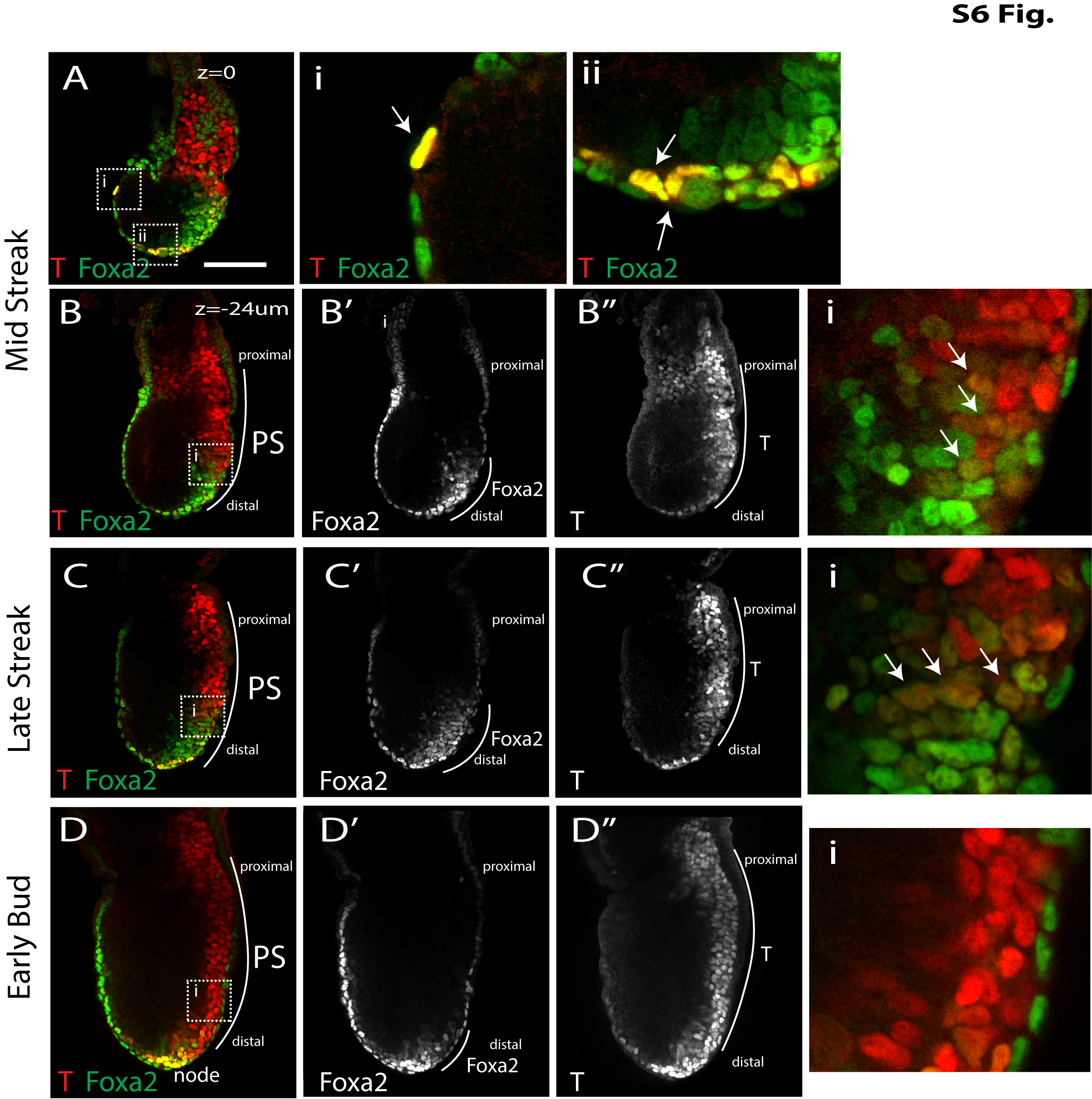

Supplement: S6 Fig — (A–D) Single optical sections from same embryos as shown in Fig 4. E6+21h MS (A–A”) and LS (B–B”) and E7+7h EB (E, F) embryos are immunostained for T (red) and Foxa2 (green). Views are lateral/slightly posterior. Insets in Ai, Aii, Bi, and Di show magnified views (A–C). White arrows point to T+/Foxa2+ double positive cells in the definitive endoderm (Ai, Aii) at MS position in MS-LS embryos (Bi and Ci). Scale bar: 100 μm. EB, “early bud” stage; LS, late-streak; MS, mid-streak; PS, primitive streak. (TIF) [file pbio.3001200.s006.tif]

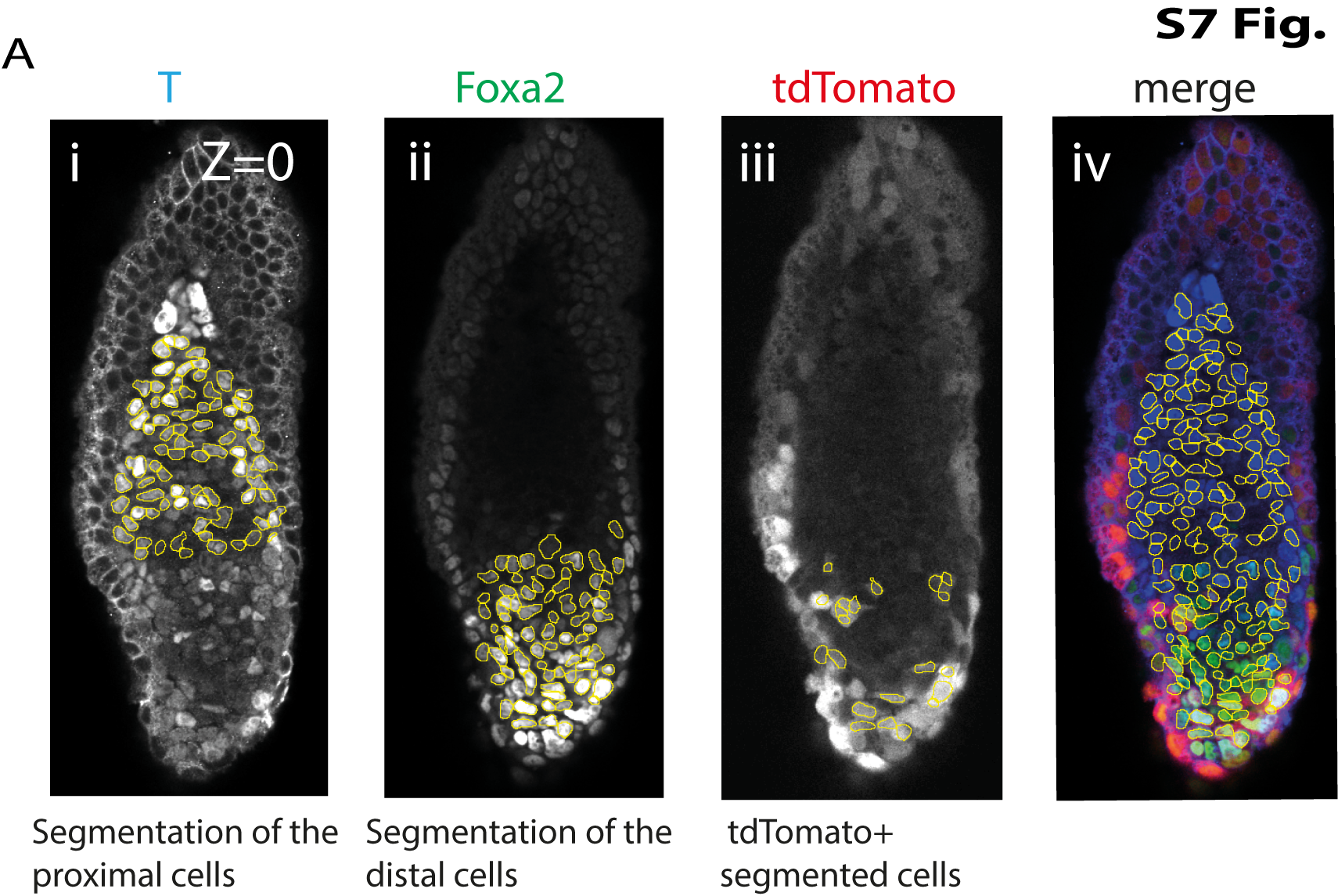

Supplement: S7 Fig — (A) Example of a segmented images based on T signal for the proximal cells (i) and Foxa2 signal for the distal cells (ii). Segmentation for only the tdTomato-positive cells is shown in (iii). Merge of the 2 segmented images (i and ii) is shown in (iv). (TIF) [file pbio.3001200.s007.tif]

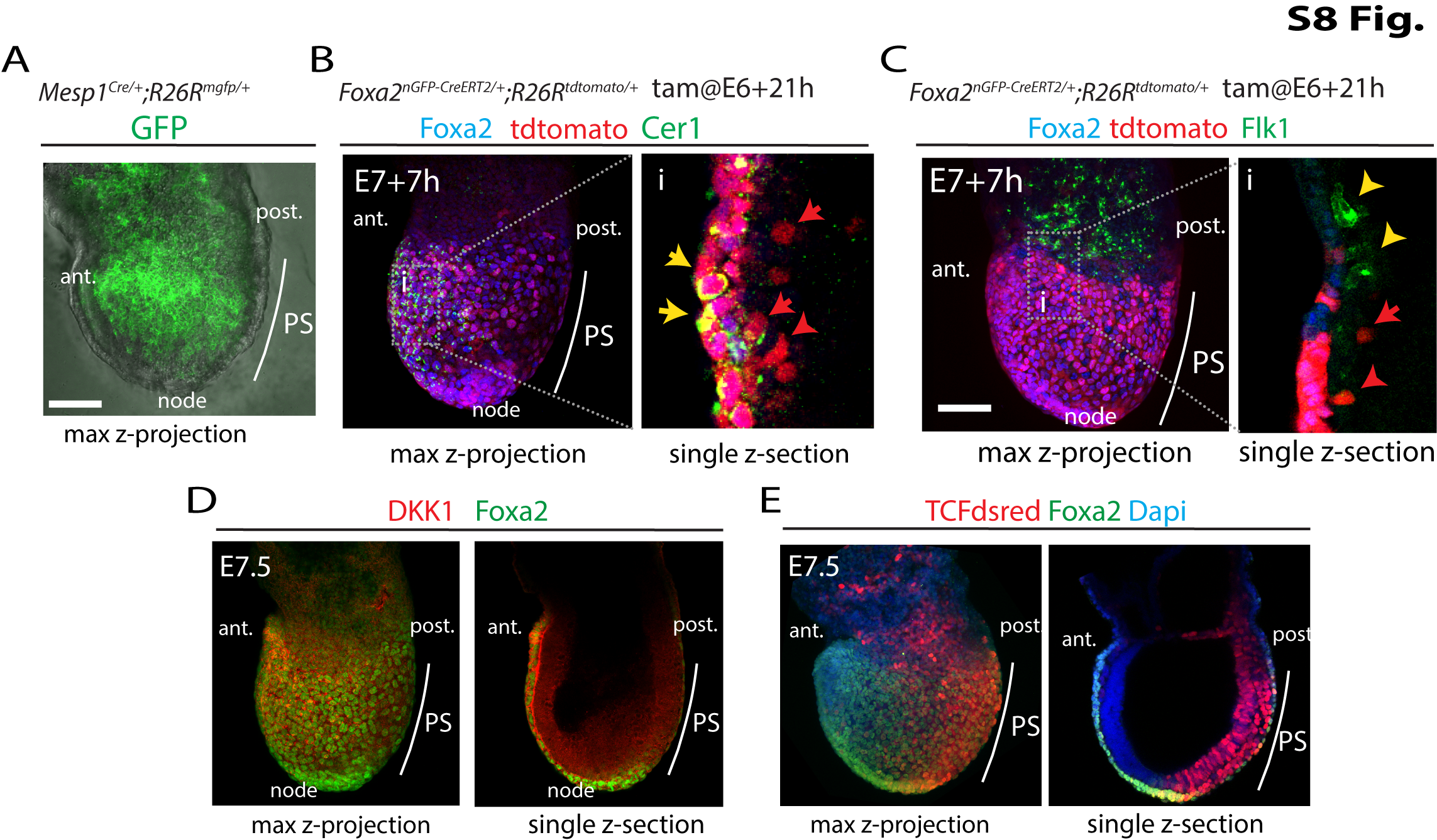

Supplement: S8 Fig — (A) Representative Mesp1cre/+;R26RmGFP/+ embryo at about E7.5. (B, C) Representative embryos resulting from the administration of tamoxifen at E6+21h in Foxa2nGPF-CreERT2/+; R26RtdTomato/+ immunostained for Foxa2 (blue) and Cer1 (blue) (B) or Foxa2 (blue) and Flk1 (green) (C). Inset in Bi–Ci show magnified view (B, C) in single optical section. (D) Representative E7.5 embryo immunostained for DKK1 (red) and Foxa2 (green). (E) Representative TCFdsred embryo (red) at E7.5 immunostained for Foxa2 (green). Ant, Anterior; post., posterior; PS, primitive streak. Scale bar: 100 μm. (TIF) [file pbio.3001200.s008.tif]

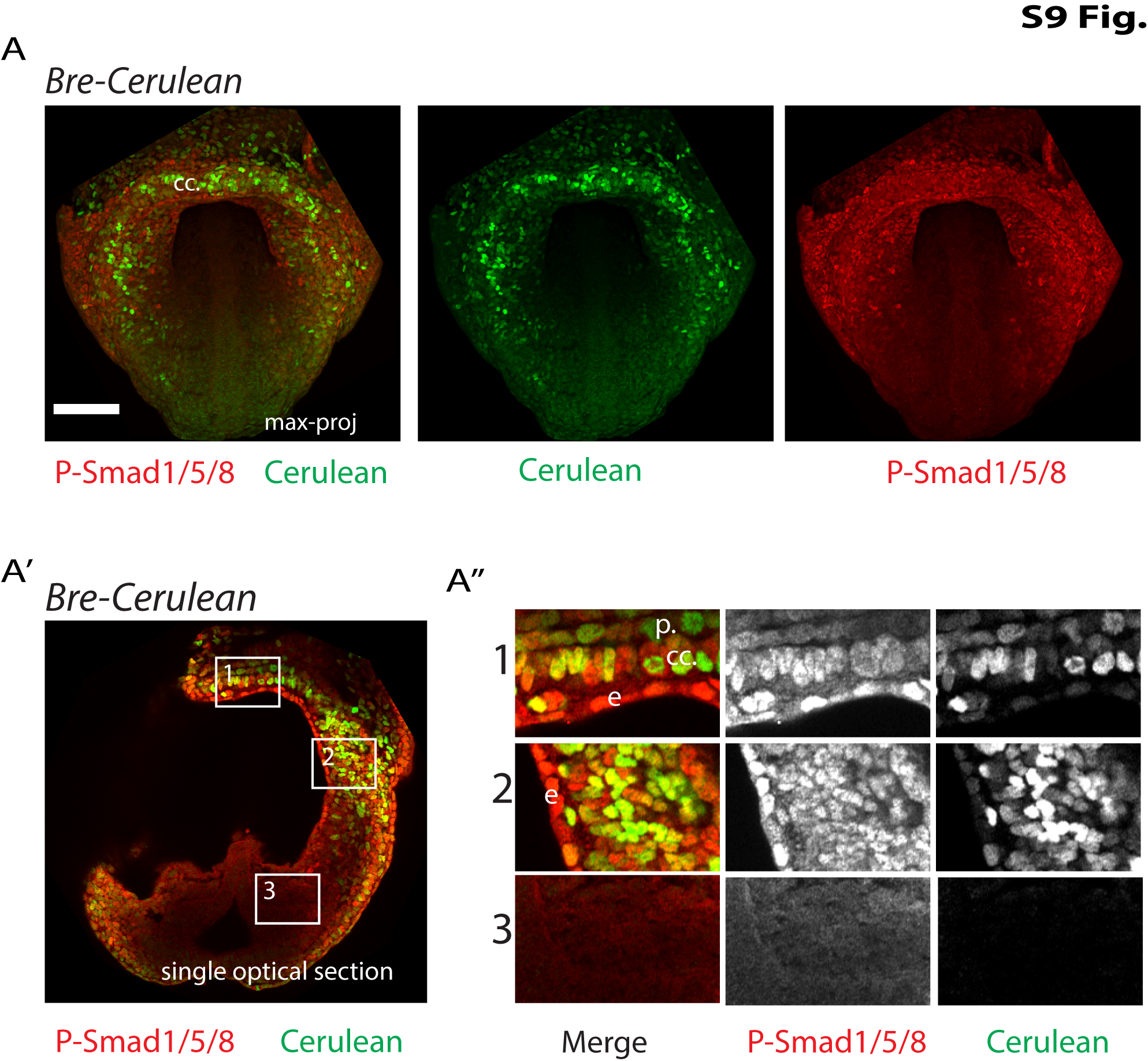

Supplement: S9 Fig — (A–A”) Colocalisation of the Cerulean signal and P-Smad1/5/8 in Bre-cerulean embryos at the cardiac crescent stage. (A) z-max proj. (A’) Single optical projection. (A”) Magnified view form insets in A’. cc., cardiac crescent; e, endoderm; p, pericardium. Scale bar: 100 μm. (TIF) [file pbio.3001200.s009.tif]

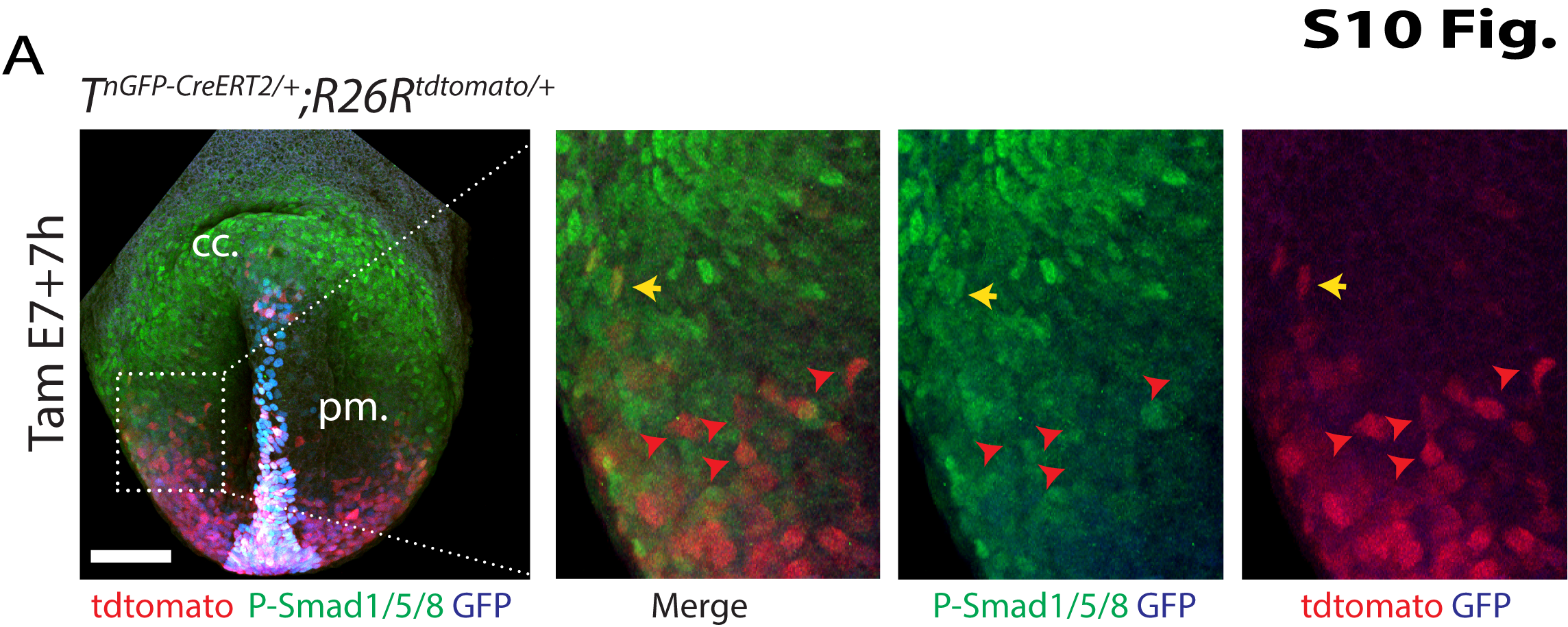

Supplement: S10 Fig — (A) tdTomato localisation in TnGPF-CreERT2/+; R26RtdTomato/tdTomato embryos immunostained against P-Smad1/5/8 following tamoxifen administration at E7+7h. cc, cardiac crescent; pm, pharyngeal mesoderm. Yellow arrow points to a Phospho-Smad1/5/8+/tdTomato+ cell, red arrows point to Smad1/5/8−/tdTomato+ cells. Scale bar: 100 μm. (TIF) [file pbio.3001200.s010.tif]

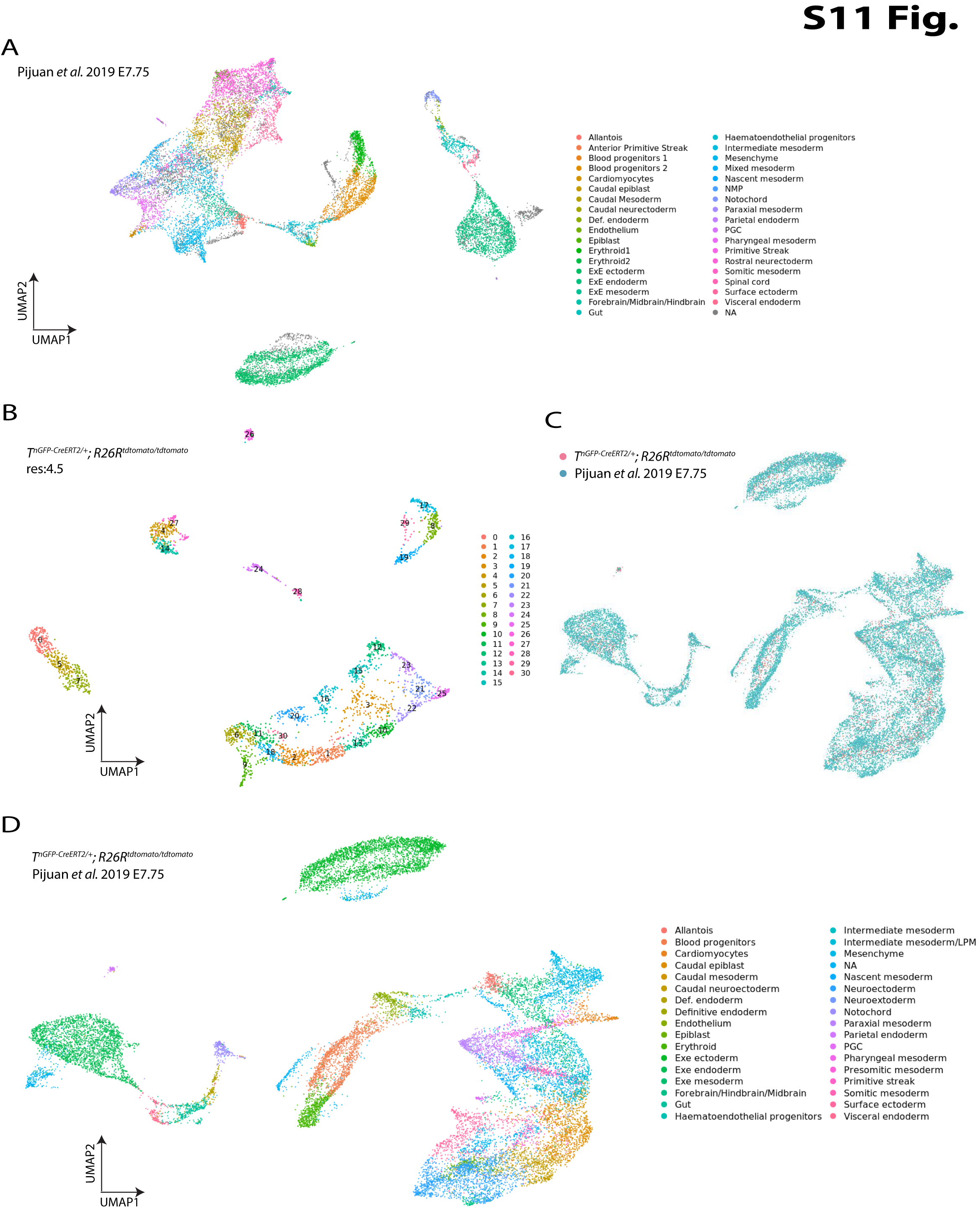

Supplement: S11 Fig — (A) UMAP plot of the Pijuan and colleagues E7.75 dataset [3]. (B) UMAP plot of the E7+7h TnGPF-CreERT2/+; R26RtdTomato/tdTomato dataset clustered at resolution 4.5. (C, D) UMAP plot showing the integrated data from the 2 scRNA-seq E7+7h TnGPF-CreERT2/+; R26RtdTomato/tdTomato and Pijuan and colleagues E7.75 dataset [3]. Colour codes correspond to the embryonic stage of collection or population identity (C) and clusters (D). Note, the paraxial mesoderm cluster is split into 2 sublcusters we named “paraxial mesoderm” and “anterior paraxial mesoderm” based on expression of marker genes (see also Fig 8D, S12A Fig, and S5 Source Data). (TIF) [file pbio.3001200.s011.tif]

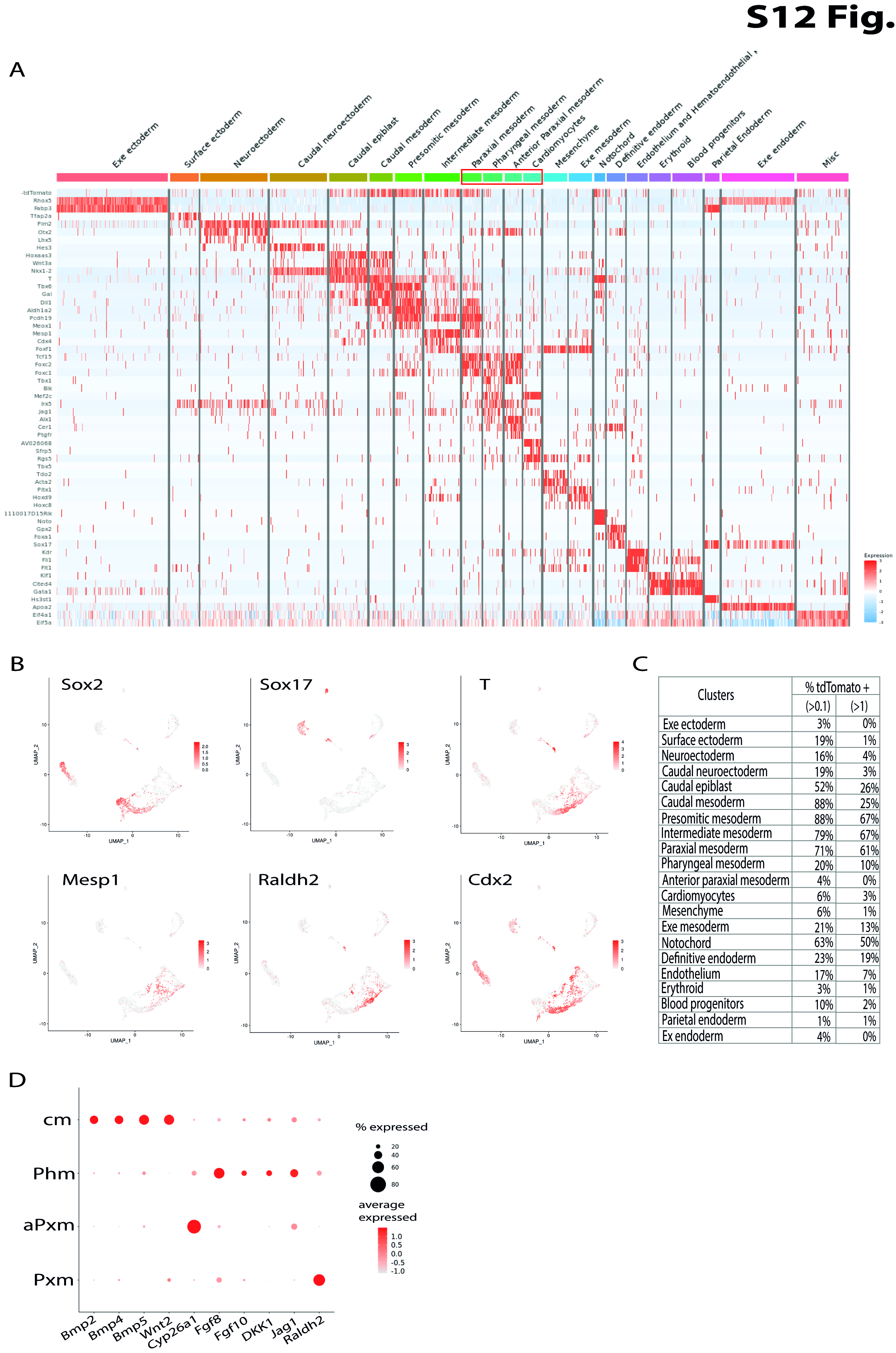

Supplement: S12 Fig — (A) Expression heat map of marker genes (S5 Source Data) and tdTomato. Scale indicates z-scored expression values. (B) UMAP showing the log normalised counts of selected genes (C) Percentage of tdTomato-positive cells in each cluster for expression values above 0.1 and 1. The data underlying (C) can be found in S6 Source Data. (D) Dot plot of factors with restricted expression in progenitors. Dot size corresponds to the percentage of cells expressing the feature in each cluster, while the colour represents the average expression level. The data underlying (D) can be found in S5 Source Data. aPxm, anterior paraxial mesoderm; cm, cardiomyocytes; Phm, pharyngeal mesoderm; Pxm, paraxial mesoderm. (TIF) [file pbio.3001200.s012.tif]

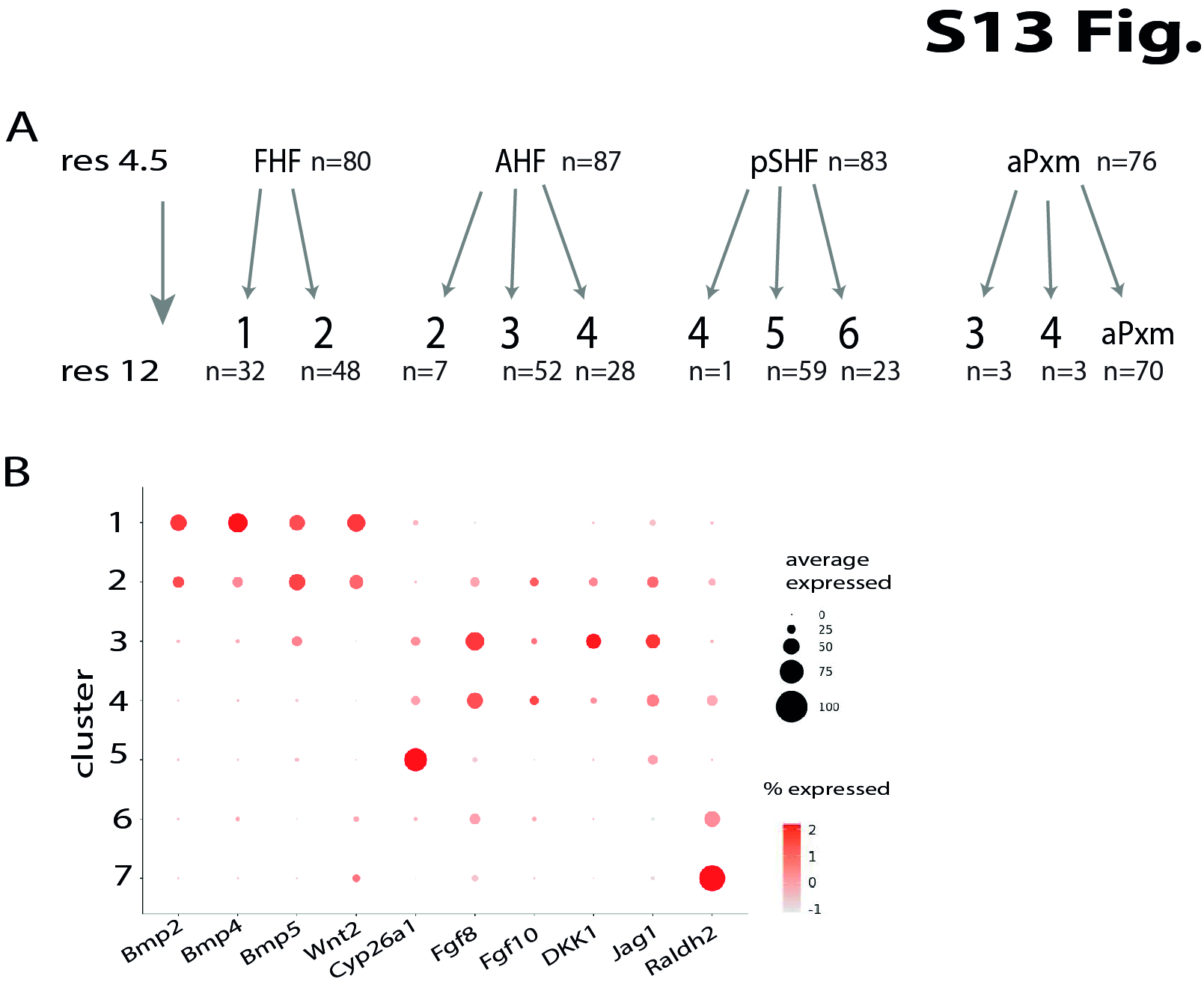

Supplement: S13 Fig — (A) Repartition of the cells from the FHF, AHF, pSHF, and aPxm cluster to subclusters 1, 2, 3, 4, 5, and 6 and aPxm. The data underlying (A) can be found in S6 Source Data. (B) Dot plot of factors with restricted expression in progenitors. Dot size corresponds to the percentage of cells expressing the feature in each cluster, while the colour represents the average expression level. The data underlying (B) can be found in S5 Source Data. AHF, anterior heart field; aPxm, anteriorparaxial mesoderm; FHF, first heart field; pSHF, posterior second heart field. (JPG) [file pbio.3001200.s013.jpg]

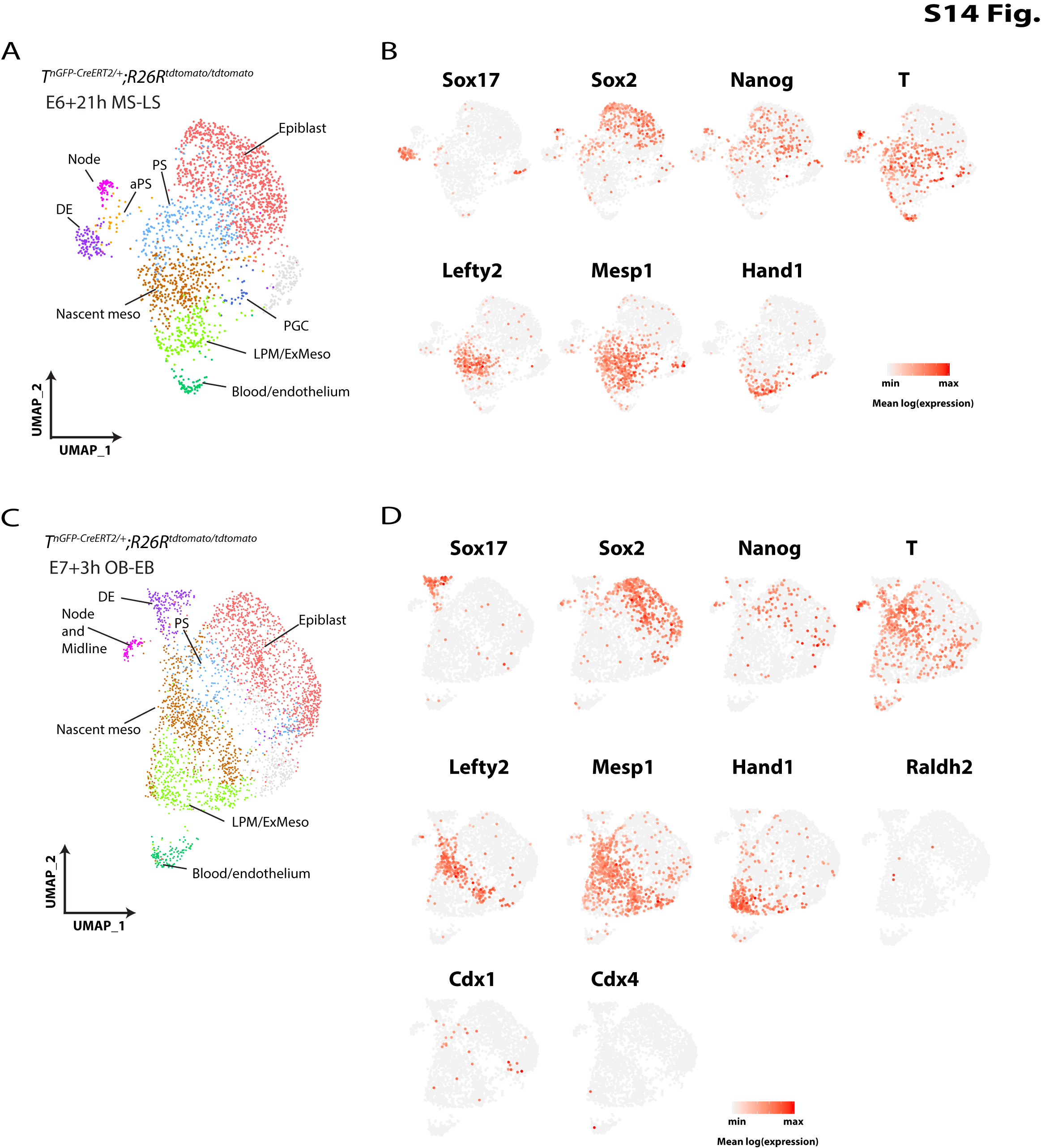

Supplement: S14 Fig — (A and C) UMAP plot coloured by cluster identity from scRNA-seq analysis of TnGPF-CreERT2/+; R26RtdTomato/tdTomato embryos at the E6+21h, MS-LS stages (A) and at the E7+3h, OB-EB stages (C). (B and D) UMAP showing the log normalised counts of selected genes. Colour intensity is proportional to the expression level of a given gene. aPS, anterior primitive streak; DE, definitive endoderm; EB, early bud; LPM/Ex-meso, lateral plate mesoderm and extraembryonic mesoderm; mesenchyme; LS, late streak; MS, mid-streak; Nascent meso, nascent mesoderm; OB, no bud; PGC, primordial germs cells; PS, primitive streak; scRNA-seq, single-cell RNA sequencing; UMAP, Uniform Manifold Approximation Projection. (TIF) [file pbio.3001200.s014.tif]

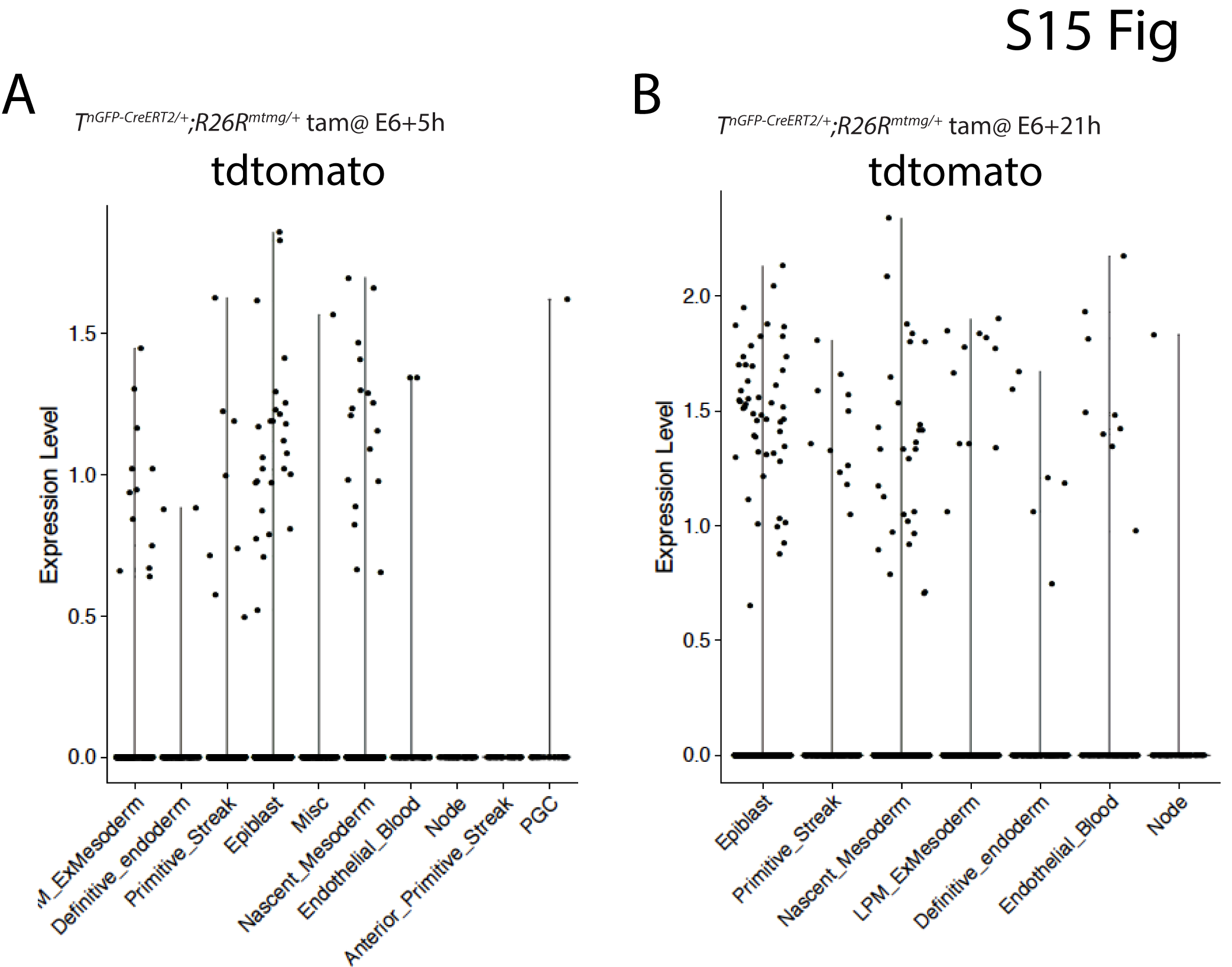

Supplement: S15 Fig — (A, B) Violin plots showing tdTomato expression for each cluster in TnGPF-CreERT2/+; R26RtdTomato/tdTomato mid-late streak (A) and OB-EB (B) embryos shown in Fig 9A. The data underlying (A, B) can be found in S7 Source Data. (TIF) [file pbio.3001200.s015.tif]

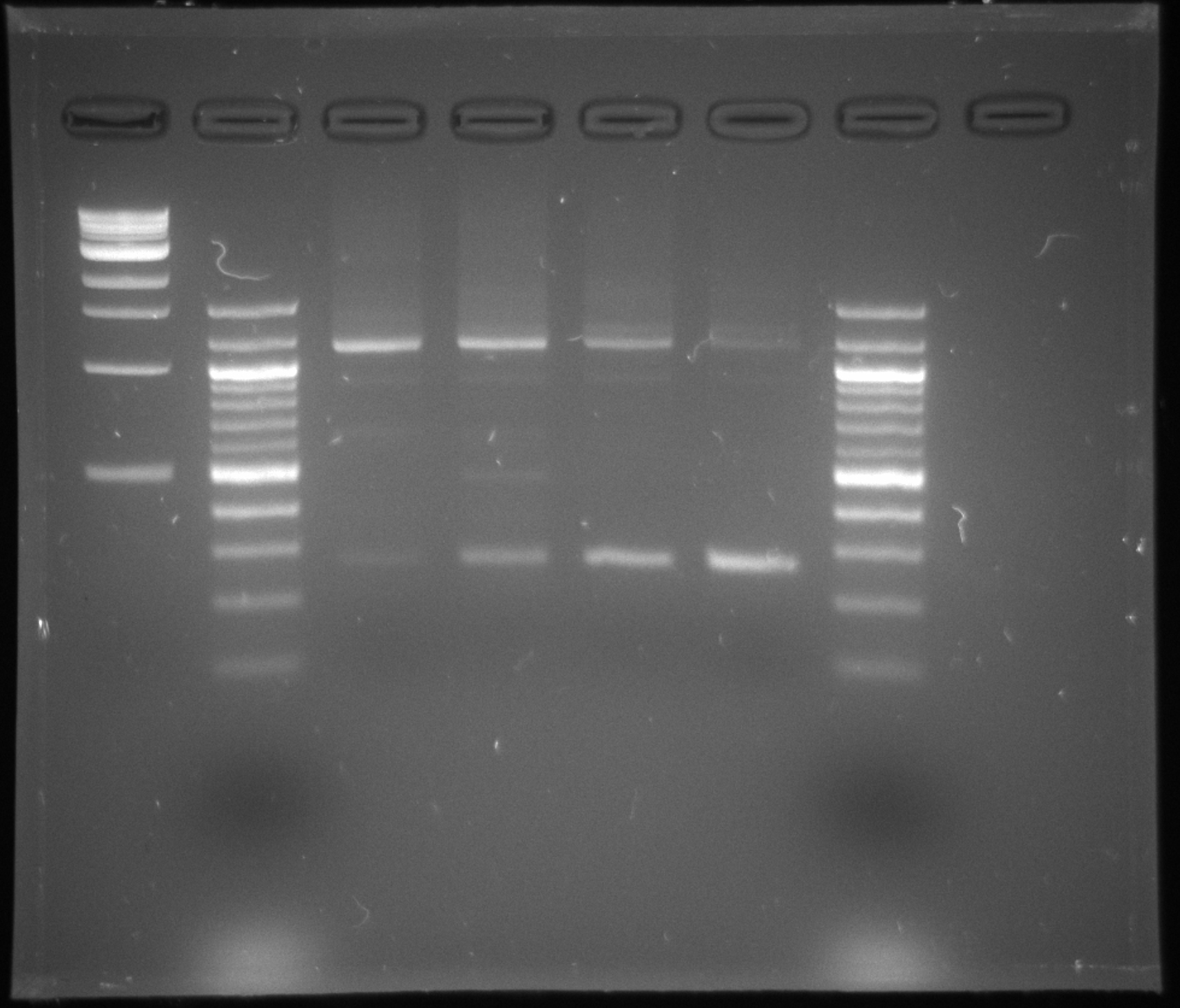

Supplement: S1 Raw image — (TIF) [file pbio.3001200.s016.tif]

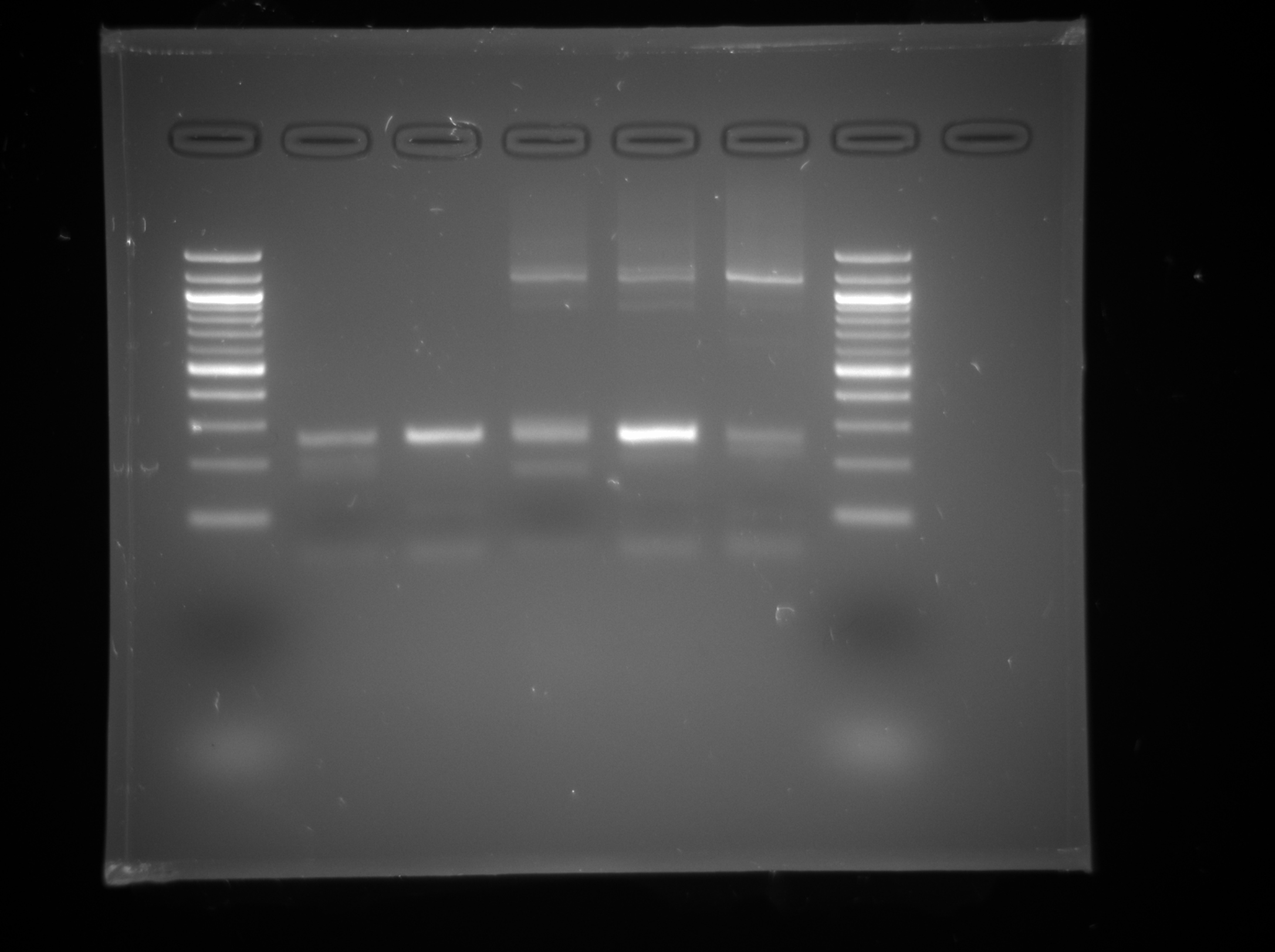

Supplement: S2 Raw image — (TIF) [file pbio.3001200.s017.tif]
