# Supplementary material for: Ventricular, atrial, and outflow tract heart progenitors arise from spatially and molecularly distinct regions of the primitive streak
Source: PLoS Biol. 2021 May 17;19(5):e3001200. doi: 10.1371/journal.pbio.3001200 (PMC8158918; doi:10.1371/journal.pbio.3001200)
Supplement: Attachment — Submitted filename: Ivanovitch et al. Response to Reviewers.docx [file pbio.3001200.s026.docx]

Dear Dr Ivanovitch,

Thank you very much for submitting your manuscript "Ventricular, atrial and outflow tract heart progenitors arise from spatially and molecularly distinct regions of the primitive streak." for consideration as a Research Article at PLOS Biology. Your manuscript has been evaluated by the PLOS Biology editors, an Academic Editor with relevant expertise, and by several independent reviewers.

In light of the reviews (below), we will not be able to accept the current version of the manuscript, but we would welcome re-submission of a much-revised version that takes into account the reviewers' comments. We cannot make any decision about publication until we have seen the revised manuscript and your response to the reviewers' comments. Your revised manuscript is also likely to be sent for further evaluation by the reviewers.

We expect to receive your revised manuscript within 3 months.

Your revisions should address the specific points made by each reviewer. Having discussed these comments with the Academic Editor, we think you should address the reviewers' concerns with more data where possible. However, we recognize that the requests of reviewer 3 are probably beyond what can reasonably be achieved in a normal revision period. Please also assess carefully which of the reviewers' concerns might be addressed by softening conclusions or discussing alternative interpretations of the data. In these cases, please provide a clear justification in the rebuttal on why the requested experiments are not essential for the main message of the paper.

REVIEWS:

*Reviewer #1: This is a well-executed and interesting study by Ivanovitch and colleages combining genetic lineage labelling with single cell sequencing of cardiac progenitors in the mouse. They performed an extensive analysis of genetically labelled cardiac progenitors emerging from the primitive streak at different times. To determine the molecular signatures of these cells, they perform single cell sequencing. High resolution clustering identified subpopulations of the FHF, AHF and SHF. Their data suggests that cardiac progenitors are already molecularly distinct as they gastrulate, which the authors suggest might be due to exposure to spatio-temporally distinct signalling environments.*

*The paper is well-written and clear with high quality data and figures.*

*Minor changes:*

*Figure 3: it would be helpful to indicate with a line the extent of the PS, and the location of the node and proximal/distal PS*

We have now annotated Figure 3 (now Fig 4).

*The description of Fig. 4 in the text and the figure legend is a bit confusing: (236) We next administered tamoxifen in Foxa2nEGP-CreERT2/+ R26Rtdtomato/+ embryos at E6+21h and fixed the embryos 10 hours later. - perhaps add = E7+7h to make it consistent with the legend, which says: (891) Localisation of the tdtomato+ cell from the Foxa2 lineage are assessed at (i) E7+7h.*

Thank you, we have changed the text to make it consistent with the legend.

*(244) Typo: (yellow arrows in Fig 2Aiv) should be Fig 4Aiv*

Thank you, we have corrected the text.

*Figure 5A, additional labelling of embryos and view displayed would help to orientate the reader, panels Aii and Aiv are not described in legend (arrows) and not mentioned in the main text*

We agree with the reviewer and we have now indicated the embryonic axis and the views displayed (now Fig7). We have now commented on the panels Aii and Aiv.

*Also comment on Figure 6D in the main text*

We have now commented on Figure 6D (now Fig 9D).

*Line 278/9, incomplete sentence: Within these subpopulations, cardiac progenitors belonging to the previously defined FHF, AHF and pSHF.*

Thank you. We have now completed the sentence describing how we have assigned FHF, AHF and pSHF identities to our clusters.

*Reviewer #2: In this manuscript by Ivanovitch et al., the authors interrogate the model that the primitive streak harbors spatially distinct cardiac sublineages pre-determined for the FHF and SHF structures. They cleverly make use of genetic lineage tracing from T and Foxa2 expressing cells to label emerging mesodermal progenitors in order to perform microscopy and scRNA sequencing analysis during gastrulation. The central thesis is that spatiotemporal segregation of these progenitors at early stages underlies later cardiac fate specification, concluding that the left ventricular progenitors are the first to exit the primitive streak, followed second by the right ventricular progenitors, and thirdly the outflow tract and atrial progenitors, which also arise from different regions. Although previous data (Lescroart et al 2014, Devine et al 2014) is consistent with the these findings, the authors do advance knowledge by demonstrating a graphic map of cardiogenesis.  The scRNAseq data and analysis, although insufficient to stand on their own, are a suitable complement to the lineage tracing data, and provide some molecular evidence for temporal (though not spatial) segregation of mesoderm progenitors.  Revision work should focus on clarifying the authors' scientific reasoning process (there are places in the manuscript where the strength of the stated conclusions is excessive for the level of evidence presented), and on providing better commentary and discussion to address how the findings fit into the larger scientific field.*

*Major Points*

*In figure 1 the authors showcase an ordered contribution of cells exiting the primitive streak into LV, RV, and OFT and atrial progenitors using single pulse labeling with tamoxifen of the T lineage at early post-gastrulation stages of embryonic development. While embryonic labeling and tamoxifen/Cre labeling kinetics were well vetted and the image analyses supports the authors' claims, the authors rely on sparse labeling of LV cells from their earliest timepoint as the primary explanation for why LV progenitors are the first to exit the primitive streak. The authors could address other explanations beyond early exit from the PS for why LV progenitors are not being captured by T lineage tracing to strengthen their claims,*

We thank the reviewer for this comment. We have now administered tamoxifen at an earlier stage (*i.e.,* E6+8h, before or at the onset of gastrulation) and we found that LV and RV progenitors were captured with similar efficiency under this condition (Figure 1Ci, D and E, LV: 31.4.2% ±12.4; RV: 31.7.2% ± 15.5, mean ± SD, P-value: ns, n=6). These data confirm our initial explanation. When tamoxifen is administered at E6+21h and E7, the LV progenitors are not being captured, we interpret this to indicate that they have already exited the primitive streak by these stages.

We have also quantified the level of T and Cre protein expression in *T^nEGP-CreERT2/+^* embryos to address why the left and right ventricular progenitors were captured less efficiently than the OFT/Atria progenitors. We found lower levels of T (and nGFP) protein expressed in ventricular progenitors (PS at the MS-LS stages) compared to the outflow tract and atrial progenitors (PS at the OB-EB stages, Fig 1J, K, L and M and J and S4Ai-ii). This may have resulted in a lower level of Cre expression in ventricular progenitors and could explain the lower recombination efficiency observed in these cells compared to the OFT/Atria progenitor pool.

*and overall some of the lineage mapping arguments should be enhanced or revised, particularly with respect to their strong conclusions about the spatial separation of progenitors during and before gastrulation. For example, in Figure 3, the authors examine spatial patterns for expression of T+ and Foxa2+ cells and conclude from whole-mount immunostaining that a previously uncharacterized population of double positive cells exists at the distal tip of the PS, near the node, and in the definitive endoderm. They assert that these are ventricular CM progenitors on the basis of similar timing for tamoxifen lineage labeling between T-CreERT2 and Foxa2-CreERT2. The authors could be more clear here about what is speculative and what is conclusive from their imaging data with regards to claims about ventricular vs. atrial lineage contributions. The graphic model in Figure 7 holds that ventricular and outflow tract CMs derive from anterior primitive streak separately from atrial CMs.  However, there are some logical inconsistencies with concluding that ventricular CM progenitors strictly originate from the anterior primitive streak:*

*1. since only 50% of the cardiac crescent is made up of Foxa2 progeny (Bardot et al 2017), it is conceivable that a proportion of ventricular CM progenitors originates from a region other than the distal primitive streak;*

This is a very important point and we have now rewritten our manuscript to convey that the distal primitive streak likely contributes to only a portion of the whole pool of ventricular progenitors (first paragraph of the new discussion). Consistent with this idea, we have now found that the Foxa2 progeny preferentially populate the middle regions of the cardiac crescent and not its more lateral aspect (Fig. 6B). The more middle/posterior regions of the primitive streak are likely to contribute to the ventricular myocardium. Importantly, this is consistent with the previous fate mapping from the laboratory of Patrick Tam (Kinder et al. 1999) showing that the distal and the more posterior/middle regions of the primitive streak at the mid-streak stage contribute to the cardiac crescent and heart tube (but not the most posterior aspect of the primitive streak that contributes instead to the allantois).

*2. in published single clone analysis of mesoderm progenitors (Devine et al 2014), many ventricle-atrium clones are noted, suggesting that ventricular and atrial CM progenitors may originate from similar primitive streak locations;*

We thank the reviewer for this important comment. As mentioned above, it is indeed likely that more posterior-middle regions of the primitive streak (Foxa2 negative) contribute to the ventricle. Perhaps these posterior cells become allocated to the proximal primitive streak at the EB-OB stages and contribute to the atrium instead of the OFT. This would account for the ventricle-atrium clones reported in the Devine et al. paper and will also be consistent with our *T*- lineage tracing. We have now mentioned the observations of ventricle-atrium clones in the Discussion.

*3. all myocardium is derived from Mesp1-lineage progenitors (Saga et al 2000 and many others), but at the time of birth of LV progenitors ~E6.5, the anterior streak is actually devoid of Mesp1 (see present manuscript Figure 6B cluster aPS, also ISH examples in Cunningham et al 2017 Figure 1, and Saga et al 2000 Figure 1).*

We now provide additional analysis of the *Mesp1*-lineage progenitors at the E7+7h stage and found, as previously documented in numerous articles, that all anterior mesoderm – *i.e.,* including mesoderm descendants from the aPS and axial mesoderm – express Mesp1 (S8A Fig.). Mesoderm derived from the aPS must therefore expresses *Mesp1*, if not initially in the primitive streak, at least during migration. Consistent with this idea, *Mesp1* is expressed in the nascent mesoderm (cf. our single cell analysis and others and ISH examples). Rare Foxa2/Mesp1 double positive cells have been reported in middle regions of the primitive streak in Probst et al 2020 (Figure3 H and I). Cells that co-express Mesp1 and Foxa2 in the aPS may therefore contribute to the ventricular myocardium. More refined lineage tracing experiments will be required to assess if those co expressing cells are ventricular progenitors.

*If there is better evidence for an aPS origin for these structures, particularly derived from Figure 3 or 4, the logic should be described in the manuscript text more explicitly. An alternate (and more modest) explanation is that the RV and LV progenitors emerge from proximal/posterior PS, since posterior epiblast transiently ("accidentally") expresses Foxa2 as the streak extends, and production switches from endoderm to mesoderm (Probst Biorxiv 2020, Figures 2 and 3).*

We agree with the reviewer that we did not provide sufficiently explicit evidence of an aPS origin for the ventricular myocardium.

We have now included additional evidence:

1) As mentioned above, previous fate mapping analysis has shown that the anterior and middle regions of the PS at the midstreak stage contribute to the cardiac crescent and heart tube (Kinder et al 1999). The aPS corresponds (approximately) to regions where cells co-expressing Foxa2 and T positive expression are detected (compare Figure 3A, Kinder et al 1998 with Figure 4A in our manuscript).

2) As mentioned above, we found that the Foxa2 descendants, including the ventricular progenitors, are all preferentially located in medial regions of the mesoderm (Fig 6A-B). These results are consistent with the observation, obtained from numerous fate mapping analysis, that the primitive streak is organized such that anterior aPS (regions giving rise to more medial/axial descendants than the posterior regions) contribute mainly to lateral/ventral tissue types. Further evidence supporting this conclusion in the mouse and for cardiac progenitors can be found in Kinder et al 2001 (Fig 3).

3) We have tested the alternative possibility proposed by the referee that the RV and LV progenitors emerge from posterior epiblast transiently expressing Foxa2 at an earlier stage and relocating in the posterior PS at the mid streak stage. We found that this was not case, since when we administered tamoxifen in *Foxa2^nEGP-CreERT2/+^* *R26R^tdtomato/+^* embryos earlier (*i.e.* at E6+8h), all the recombined tdTomato were located in the Foxa2 positive anterior domain of the primitive streak at the mid streak stage (new Figure 5). We conclude that cells expressing *Foxa2* at early timepoints maintain *Foxa2* expression in the anterior primitive streak as they switch contribution from endoderm to the left and right ventricles. This rules out the relocation of cells with a Foxa2 lineage to the posterior PS at mid-streak stages. We have added a paragraph in the result section.

*In figure 2 the authors utilize Foxa2 lineage tracing to investigate origins of ventricular vs. atrial myocardium cells. Interestingly, labeling with Foxa2 at the same early timepoint of T lineage tracing yielded labeling of both LV and RV myocardium (line 198-201) however lineage labeling at the same stage with T did not produce robust labeling of LV cells in figure 1. In both figures the lineages look pretty sparse but the interpretation discrepancy is not elaborated on in the authors' conclusion that Foxa2+ primitive streak cells contribute to the ventricular myocardium at similar stages to T+ cells (line 203-204).*

We agree with the reviewer and we have now quantified the proportions of the tdtomato+ cardiomyocytes populating the LV and RV when tamoxifen was administered at E6+21h in *Foxa2^nEGP-CreERT2/+^* *R26R^tdtomato/+^* embryos. We found it to be low for both LV and RV (for E6+21h, left ventricle: 9.4% ± 9.5 right ventricle: 11.2% ± 10, p-value:ns, Figure 3D- E). In 3 out of 14 heart no tdtomato-expressing cells could be detected at all in either the left or right ventricles (Figure 3D- E). And we did not observe, in contrast to *T,* a lower contribution to the LV compared to the RV. We cannot therefore conclude from results obtained with the *Foxa2* lineage tracing that the LV progenitors leave the PS first followed shortly by the RV progenitors.

Several reason can explain the low efficacy of recombination in both LV and RV progenitors. First, the aPS expresses low level of *Foxa*2 (Figure 5C-F). Second, Foxa2 expression is likely to be more transient than *T*, since it is rapidly downregulated in the primitive streak when cells become mesoderm and *T* is expressed for longer period of time and is also detected in the nascent mesoderm (Fig 9B). Finally, as mentioned above, the *Foxa2* progenitors are likely to represent only a subset (50%, Bardot et al. 2017, the maximum contribution we observed in our analysis for both LV and RV is ~30%) of the whole pool of the ventricular progenitors. By comparison, we found the majority of the definitive endoderm (robustly expressing *Foxa2)* to be populated by tdtomato positive cells (Fig 6A-B).

*Similarly with the above, it is not conclusive that atrial progenitors necessarily arise from proximal primitive streak (lines 251-2) based on absence of Foxa2 in that location, since it is conceivable that they arise distally but are just Foxa2 negative.  To draw this conclusion definitively requires that all mesoderm derivatives (or at least those destined for the cardiac crescent) from distal PS are also Foxa2-positive or Foxa2-lineage-positive.  If the authors use Figure 3 to support this claim, the logic should be described in the manuscript text more explicitly (lines 247-9).  It might be helpful to have orthogonal view projections or slices (axial or coronal) to accompany 3A, 3C, or 3E, because this would show the different primitive layers.*

We agree with the reviewer and we have now clarified this point in our manuscript. Our new quantification shows that all aPS cells express Foxa2 at a low level (Fig 5F). This suggests that all aPS cells have a low probability to recombine. Considering that in our *Foxa2* lineage trace (and from others, Bardot et al.) contribution to the atria is never observed, we deduce that the distal PS (*Foxa2* lineage positive) does not contribute to the atria. The atria progenitors must instead be arising from a more proximal location in the PS (Foxa2 lineage negative).

We have now also provided the axial slices to accompany Fig 4 in S6 Fig.

*The authors use figure 4 to illustrate the potential for a stream of migratory cells (distal PS to anterior embryo) corresponding to presumptive cardiac crescent. Addition of known marker genes and a more clear distinction between speculation and definitive conclusions could aid in conclusions drawn from this figure.*

We agree. We have now better characterised these cells by adding more markers (Figure 6B and S8). We found some of these cells were responding to BMP signalling (labelled by the BMP reporter Bre-cerulean, Fig6 B). They also have characteristics of cranial mesoderm progenitors as they are located adjacent to the anterior endoderm expressing the Nodal antagonist Cer1 and Wnt antagonist DKK1 (S8B and D Fig) while the PS and posterior mesoderm is instead marked by Wnt/bcatenin signalling activity (S8E Fig). We found no evidence that these cells were endothelial cells since none of them expressed Flk1, a well-known endothelial marker (from n=7 embryos analysed at E7+7h, S8C Fig).

*In figures 5 and 6 the authors utilize scRNAseq to interrogate molecular signatures of RV, OFT, and atrial cardiac progenitors within their T lineage labeled embryos, and to further support the distinction between progenitor pools. The analysis is curated to support the temporal segregation of progenitors story told in earlier figures with the microscopy data, although the authors could make the complementation of the findings more clear in their text to strengthen their spatial and temporal conclusions shared in this section of the manuscript. The authors also make use of published datasets to understand subpopulations within their own data, and could elaborate more on their methods for doing so. Additionally, more explanation for how cell types were labeled (what marker genes were used?) would benefit reader interpretation of UMAPs, particularly in Fig 5D.*

We thank the reviewer for these comments, and we have now revised our single cell analysis and explanations. First of all, we have added an additional sequencing run of the E7+14h embryos. After merging the two sequencing runs, the depth of sequencing has increased from 14,711 reads per cell to 47,975 reads per cell. The reanalysed data provides greater resolution but has not changed the overall conclusion.

We now explain the methodology we used in the Material and Methods section and with a new supplementary figure that indicates how we made use of the published datasets (S11 Fig). In short, we have integrated our E7+14h dataset with the E7.75 dataset provided by Pijuan et al 2019. This allowed us to assign identities to our cell clusters. In particular, we assign a pharyngeal mesoderm, cardiomyocytes, anterior paraxial and paraxial mesoderm identities to our cells. Based on marker gene expression provided by Lescroart et al. for the cardiomyocytes, AHF and pSHF (at earlier stages ~E7.27), we could further assign cardiomyocyte, AHF and pSHF identities. The marker genes for cardiomyocyte were: Tbx5, Hand2 and Tbx20; for AHF were: Tbx1, Fgf8 and Fgf10 and for pSHF were Raldh2, Tbx6 and Hoxb1 (see feature plots for each of these genes and corresponding clusters in Fig 8C).

*Interestingly, as pointed out above, expression of Mesp1 is undetectable in the aPS population relative to other identified regions in fig6B - could the authors explain their interpretation of this finding in relation to referenced published works from Lescroart and Devine wherein Mesp1 lineage is shown to contribute to heart?*

We agree with the reviewer. We have already addressed this point above.

*Additionally, Hand2 and Isl1 in Figure 5E are SHF markers - why are these genes highly detected in the cluster labeled "LV" relative to RV, OFT, and atria?*

We thank the reviewer for this comment. *Hand2* is indeed detected in the cardiomyocyte cluster at E7.5. The Lescroart data show a similar expression profile for Hand2. Moreover, ISH also shows *Hand2* to be expressed in anterior location where the cardiomyocytes emerge at E7.5 stage before becoming restricted to the SHF (Souihol et al. Development 2015 142: 3649-3660, Figure 7). Similarly, Isl1 is expressed transiently in the cardiac crescent (Prall et al 2007 Figure 3 and Ma et al. 2008 doi: 10.1016/j.ydbio.2008.08.013) before becoming restricted to the SHF. This transient expression of Isl1 in the cardiac crescent explains why sparse recombined cells can be identified in a population of left ventricle cardiomyocytes in Isl1 cre based lineage tracing (Cai et al. 2003). We have added the feature plots for Hand2 and Isl1 to Fig 8C and G.

*The authors could also provide more methods explanation for the discrepancy between ages of embryos analyzed via microscopy (generally a few hours older) and embryos analyzed via scRNAseq (generally a few hours younger),*

We agree with the reviewer that we needed to clarify our staging for embryos analysed via scRNAseq. In particular for Fig 9, we analysed four embryos from a litter collected at E7+3h instead of E7+7h for the microscopy experiments (Fig 6A). However, what was important is the embryonic stage of the embryos analysed via scRNAseq (OB-EB stages in this case since this is the stage when the OFT and Atria progenitors are exiting the PS). We have clarified in our revised manuscript by referring to embryonic stages rather than by the time the embryo were collected.

*as well as more notes on the methods and interpretations for performing the cell type and tdTomato lineage labeling in their scRNAseq analysis. The tdTomato expression was compelling in the microscopy images - the authors could comment more on the trends they observe with the lineage marker within the scRNAseq data.*

We thank the reviewer for this comment. We have now provided the percentage of tdtomato+ cells for each of the cell cluster (Fig 8Diii, S12C Fig and S8-source data). We have also commented in the Results section of the trend we see in the scRNA seq data.

*It was difficult to determine which conclusions were firm and which were more speculative in this section of the manuscript, especially with regards to the signaling gradients discussed.*

We have now provided a dot plot showing expression level and percentages of the cells expressing BMPs, Fgfs, Wnts, Cyp26a1 and Raldh2 in each of the cardiac progenitors/anterior paraxial mesoderm clusters (S13B Fig).

*Overall the scRNAseq computational methods should be better explained.  For example, the authors draw from prior published scRNAseq datasets, but do not describe any specifics that contributed to their cluster assignment (lines 278-9 contain an incomplete sentence).*

We agree with the reviewer and we have added these descriptions (see above and S11 Fig).

*Fig 5C-F is particularly problematic, as it assigns anatomical futures to groups of cells. This is not appropriate and needs to be revised to reflect simply that there are different clusters at E7.5, some of which contain tdTomato.*

We have now removed any reference to anatomical features to groups of cells and renamed the clusters with numbers.

*The use of the tam@E6+5h in Figure 6 isn't well explained, especially since this time point is not represented in Figure 1 - unless detailed further, the differential gene expression could appear to result from embryo vs. extraembryonic mesoderm differences (lines 342-344), not ventricular vs. outflow tract progenitors (lines 357-9, 360-1). The re-clustering process used to generate this figure should be explained better or overtly indicated in the figure. The relationship of tdTomato-expression to the clusters show in 6E-H is needed to help clarify this. The tdTomato lineage is not shown or described in Fig 6, making this figure no more than an incomplete recapitulation/reanalysis of published data, and therefore without a link to the previous data should be removed.*

We agree with the reviewer that we did not explain why we use the tam@E6+5h in Figure 6. The rationale behind this experiment was to distinguish the embryonic tdtomato negative embryonic mesoderm (contributing to cardiac) from the tdtomato positive extraembryonic mesoderm emerging from the early primitive streak. Unfortunately, this turned out not to be possible since there were too few tdtomato reads in these datasets, probably due to the lower number of genes detected compared to the previous E7+14h -EHF stage- dataset (S15 Fig). The use of the tam@E6+5h is therefore not relevant for the analysis (*i.e.* comparing the PS cells at the MS-LS vs OB-EB stages) and we have clarified this in the text.

*As with Figure 5, there is no new information to help understand the spatiotemporal experiments presented with the lineage tracing.*

As mentioned above, we have now documented the percentage of tdtomato positive cells in posterior versus anterior mesoderm. We think this will help a reader connect the molecular and lineage tracing data.

*Overall, the data presented is of high quality, and provides a framework for a manuscript that will be impactful. To help this paper convey its message more clearly, the authors should focus on providing better explanations of the methods and observations, in addition to re-writing conclusion statements to better account for alternate possibilities.*

We thank the reviewer for their constructive suggestions. We have now clarified the methods and observations, added additional experiments to address the alternative possibilities, and rewritten some of the conclusion statements.

*Minor points*

*How did the authors decide how many embryos were sufficient for their imaging conclusions?*

We agree that the number of cases to support that independent Foxa2 progenitors for LV and RV exist is low. To obtain more embryos will require redoing lineage tracing with lower doses of tamoxifen. We however maintain that these results show independent Foxa2 progenitors for LV and RV exist. We do not rule out the possibility of a common progenitor.

We have now also provided p-values for Fig 1 and 3 (in the text) The p-values support our conclusions that there is equal contribution to the LV and RV when tamoxifen is administered at E6+8h and a lower contribution to the LV compared to the RV when tamoxifen is administered instead at E6+21h and E7. The p-value is also consistent with our conclusion that no differences in contribution to the LV and RV can be deduced from the Foxa2 lineage trace experiments.

*To what depth (reads/cell) were the analyzed scRNAseq libraries sequenced?*

We have added the information in the Material and Method section

E6+21h: 32,629 reads/cell

E7+3h: 64,340 reads/cell

E7+14h: 47,975 reads/cell

*Figure s6 is missing panel E as described in the figure legend*

OK

*Fig 5: showing Cerulean in green and GFP in blue is confusing*

We agree with the reviewer. Unfortunately, we found that the blue colour was not very visible. Using the green channel instead allows us to show the data with better clarity.

*Reviewer #3: In this study Ivanovitch and colleagues used combined lineage tracing, via inducible T-CreERT2 and Foxa-CreERT2 and R26R-tdTomato reporter crosses, and scRNA-Seq analyses to fate map and molecularly profile primitive streak (PS) gastrulation stages to investigate the spatiotemporal patterning of cardiac progenitors.  The authors propose that cardiac progenitors are prepatterned in the PS and this determines their contribution to distinct anatomical compartments within the forming heart. Specifically, they identified that ventricular and outflow tract (OFT) progenitors originate from the distal PS and atrial progenitors from the proximal PS and that there is a sequential contribution, such that LV progenitors emerge first followed by those of the RV and then those of the poles (OFT and inflow tract; IFT).  Single cell RNA-Seq identified these sub-populations to be molecularly distinct groups of progenitors and that discrete transcriptional programmes are established before migration out of the PS. The Foxa2 lineage tracing confirmed a common lineage between the RV and OFT within the pre-defined SHF, and supported previous clonal findings, by Meilhac et al. (2014) and Lescroart et al (2014), that atrial progenitors segregate as an independent progenitor pool.*

*This study converges on further understanding of cardiac progenitor specification and commitment in the early mouse embryo, with insight into how discrete progenitor pools and subpopulations can emerge coincidentally to contribute to distinct anatomical regions of the developing heart.  However, there are issues with the study in its current form and some over-reach on interpretation that requires further experimental data to support:*

*General comment:*

*The conclusions of the study are thus far based exclusively on lineage tracing and SCRNA-Seq. However, there appears to be an over extrapolation from two types of experiments that are only weakly linked by the RNAseq having been performed on labelled samples.  Importantly, there is a lack of validation experiments and no genetic proof of the spatiotemporal patterning described; within, and emerging from, the PS.  Two suggestions to provide important validation are as follows:*

*i) the authors should carry out some low-throughput spatial transcriptomics, ie RNA FISH, RNAScope or HCR to localise some of the specific sub-population markers to discrete stages and regions of the PS; multiplexing markers here that have emerged from each of the sub-populations defined by the scRNA-seq would serve as an important validation of the early spatially distinct specification within the PS;*

*and/or*

*ii) utilising a specific marker(s) emerging from the scRNA-seq data the authors c/should generate a Cre-driver for more refined lineage tracing of the contribution from PS sub-populations into the heart.  Alternatively, this could be used for lineage ablation crossing with a DTA-floxed mouse (available from JAX) and phenotyping, or they could target a specific marker (as validated by the FISH/RNAScope/HCR suggested above) with either of the T-CreERT2 or Foxa2-CreERT2 cre-drivers. One or more of these approaches would not only support the identification of discrete cardiac progenitor pool(s) but also the contribution to a distinct anatomical region, and would map onto the predicted insights into specific forms of congenital heart disease.*

These are nice suggestions for future work. However, we do not think that generating novel cre mouse lines nor undertaking an RNA fish RNA scope analysis are necessary since our lineage tracing data and validation are sufficient to support the conclusions we draw. The lineage tracing data provides evidence that the first progenitors to leave the primitive streak contribute to the left ventricle, shortly after right ventricle progenitor emigrate, followed by the outflow tract and atrial progenitors. In addition, we have now added further experiments in our revised manuscript to support an anterior primitive streak origin of the ventricular and OFT myocardium and a posterior origin of the Atrial myocardium (see above in our response to reviewer 2). Our single cell experiments complement the lineage tracing and show the ventricular and OFT/Atria progenitors are already molecularly distinct in the PS and that mesoderm arising from early vs late PS are also distinct.

The ablation experiment is also an interesting suggestion but out of the scope of our manuscript since these experiments will address a regeneration question rather than helping us to establish a topographic map of early cardiac development. These experiments are not feasible within the timeline of a normal revision. That said, we agree with the reviewer that our work is opening up the opportunity for more experiments and we thank the reviewer for these valuable suggestions.

*Specific comments:*

*1) The T+ and Foxa2+ lineage tracing studies are carried out exclusively utilising the single R26R-tdTomato reporter line. Whilst this is a widely used reporter, there is an acceptance that any reporter may capture a unique pattern with potential for ectopic labelling and so it would be helpful if the authors included at least some of their fate mapping analyses with an alternative reporter (such as R26R-mTmG or R26R-EYFP) to confirm and reinforce the specific labelling with each of the Cre drivers.*

We agree with the reviewer that different reporters display different efficacy of recombination and in our hands the R26tdtomato reporter is highly efficient compared to some other reporters such as the R26mtmg (Muzumdar et al 2010). The high efficiency of the tdtomato reporter was critical for our lineage tracing since our aim was to capture a maximum of progenitors expressing *T* or *Foxa2* from the time of tamoxifen administration up to 12 hours after (pulse labelling). We have now added supplementary experiments combining the *T^nEGP-CreERT2/+^* transgene with the R26^mGFP/+^ reporter instead (S2A Fig.). As expected, -since the R26^mGFP/+^ is less efficient- when tamoxifen was administered late (E7+7h) no mGFP cardiomyocytes were identified in the left ventricle myocardium. mGFP cardiomyocytes instead populated the outflow tract and atria. Conversely, an early tamoxifen administration (at E6+8h) led to mGFP positive cardiomyocytes populating the left ventricle myocardium in addition to the RV, OFT and Atria.

*This is also important when considering the reliance of tdTomato-expression to discriminate molecular profiles and subpopulations of progenitors in the scRNA-Seq analyses. This is not a request to repeat the study with an alternative reporter, simply to validate some of the initial lineage tracing*.

We have used the same R26tdTomato reporter (Madisen et al.2010) for both the imaging and the scRNAseq experiments. This was done to ensure that the tdtomato positive clusters in our scRNAseq match the tdtomato positive cells from our imaging.

*2) The T-lineage labelling experiments (Figure 1) are quite confusing and lack evidence that variations in staging of embryos account for differences in contributions to LV and RV. This c/should be tested by labelling earlier.*

We agree with the reviewer that it is important to include data from an earlier labelling. We now include an experiment where we have administered tamoxifen at E6+8h (Fig 1Ci). As discussed above in our response also to Reviewer 2, these results support the view that variability in staging at E6+21h and E7h can explain why LV progenitors are not always being captured in our T-lineage trace when tamoxifen is administered at E6+21h.

*3) Related to the lineage trace experiments, it is not clear that looking at surface labelling is a good proxy for contribution to the heart. This needs to be validated in a few hearts, combining quantification of surface labelling with disaggregation of labelled cells and flow sorting to more accurately quantify and see if they agree*.

We respectively disagree with the reviewer. Similar analysis from images in lineage trace experiments are routine in many studies in the heart (for example for the Isl1 lineage -Cai et la. 2003, Foxa2 lineage -Bardot et al. 2017, Six2 lineage, Zhou et al. 2015 doi.org/10.1016/j.celrep.2017.01.002) and in other tissues/organs. Moreover, quantification of clone size (for example from Mesp1 lineage trace-based experiments in the heart Chabab et al. 2016 Cell Rep. 2016 Jan 5; 14(1): 1–10 and in the limb…etc) has been the basis of more complex modelling aimed at inferring mechanisms of growth and morphogenesis. Analysis of the surface labelling is therefore a routine method in the field and was sufficient for the analysis of hearts from *T/Foxa2* lineage tracing and to deduce the orderly allocation of the cardiac progenitors to the heart (see Fig 1C for example). We do not consider that quantification of surface labelling with disaggregation of labelled cells and flow sorting to be more accurate.

*4) Tamoxifen administration - the experiments on tamoxifen rate of recombination are unclear. It is surprising that recombination via oral gavage occurs after 2 hours given previous studies.*

Oral gavage was performed as described in the Material and Method and demonstrate recombination with a single full dose of tamoxifen diluted in corn oil (0.08mb/body weight, Fig 1I and S4 Fig).

*Moreover, these experiments where conducted on E9.5 embryos, which does not appear to relate to any timepoints in the study. In addition, the accompanying PCR results are from pooled embryos and should be conducted on individual embryos to see the amount of recombination occurring per embryo and to monitor variation between embryos per litter.*

We thank the reviewer for their comments. In order to address this, we have performed additional PCR experiments to confirm recombination is occurring in embryos at timepoints analysed in the study – specifically, E6+21h (~mid-to late streak) and E7+7h (~OB-EB) (S4 Fig).  These data reiterate the findings of our previous experiments, *i.e.* that recombination is occurring 2 hours after tamoxifen oral gavage. Whilst we appreciate the reviewer’s suggestion of undertaking PCR on individual embryos, we do not think it will add anything to the analysis. Moreover, the purpose of this experiment was to validate that recombination starts to occur around 2h following tamoxifen oral gavage, and we believe the existing data, together with the new additional PCR results, support this conclusion sufficiently. We have also included new data in the manuscript (Fig 1H) showing CreERt2 relocalising to the nuclei two hours after tamoxifen administration at full dose. These results are consistent with the PCR results.

*Time points chosen - Why does the study use E6+21 and E7; arguably a more valid experiment would be at E6 and E6.5?*

The reason for E6+21h is purely practical. As we described in Material and Methods, mice were synchronised in oestrus and crossed over short periods of two hours from 7am to 9am. Vaginal plugs were checked at 9am and if positive the embryonic day was defined at E0. This means E6+21h correspond to 6-7am on day 7 and that was the earliest time the light-dark cycle of the facility allows an oral gavage to be administered to the mouse. Of course, what is important is not so much the timing of tamoxifen administration (E6+21h, E7, or E6+12h) but rather the embryonic stages at which the tamoxifen was administered. This is what we have documented in our analyses of litters at E6+21h and E7+7h (Fig 1F and G).

*The text is also misleading here in stating labelling was conducted from E6.5, when in fact it started at E6 +21 hours and as such, given the time for recombination, labelling is more likely from E7.25/7.5 when a large proportion of cardiac progenitors will have already left the PS(?).*

We thank the reviewer for this comment and have now changed the text. We replaced “from E6.5 to E7.5” by “successive stages of gastrulation” to avoid confusing the reader. But again, what is critical is the embryonic stages (referring to the Thelier staging system, Down and Davies, 1993) rather that the exact time tamoxifen was administered (E6+21h etc… which refer solely to the experimental method).

Of course, from DNA recombination, to RNA production, protein synthesis and protein/fluorophore maturation, more time will have elapsed. The earliest tdtomato fluorescence we could detect was 7 hours after tamoxifen administration. The signal was very faint and required long exposures (at least 1min) under the microscope to be detected. 12 hours later, the signal is much stronger. The moment the cell is “marked” however is when cells located in the primitive streak and expressing *T* and/or *Foxa2* undergo recombination.

*Also given the manuscript shows tamoxifen at E5 labels CMs why have these timepoints not been been included?*

We did not include the E5 stage because there is no primitive streak at E5, *T* is not expressed yet. E5 corresponds (approximately) to the stages the epiblast undergoes lumenogenesis. The rationale behind the E5 time point was to test the perdurance of the tamoxifen activity when administered at full dose by oral gavage. We show it does perdure since it led to the recombination of cells that express *T* ~24 hours later.

*- Labelling at E6+21 marks LV, RV and atria, if the authors include earlier timepoints, do they find a regimen that only labels ventricles and atria, ie FHF?*

We thank the reviewer for this important comment. We addressed these points in our manuscript by administrating tamoxifen at E6+8h. We did not find a regime that labels only ventricles and atria *i.e*. FHF (Fig 1Ci). Instead, we concluded that most of the atria must be derived from the late primitive streak since most of the atria are labelled when tamoxifen is administered at late E7+7h timepoint (Figure 1). Consistent with this conclusion, our new analysis revealed the late E7+7h *T* cell descendants contributing to posterior regions of the FHF and to the inflow tract of the heart tube (Figure 2). We further identified a sparse contribution of the late E7+7h T cell descendant in posterior regions of the BMP positive domain marking the FHF at earlier stages (EHF stage, Figure 7B). This suggests that late E7+7h T cell descendant are gradually recruited to posterior regions of the FHF. We also found that the late E7+7h T cell descendants were not contributing to the recently discovered juxtaposed cardiac field (JCF) (Figure 2A) (Tyser *et al.* 2021, Zhang et al. 2021). This is consistent with their finding that the JCF cells contribute mostly to the atrio-ventricular canal (AVC) and left ventricle but not to the atria (see data in table S4 at e10.5 in Tyser et al. 2021 and one rare contribution to the dorsal aspect of the atria is noted in the Zhang et al. clonal analysis (Figure 5h)). These lineage tracing/clonal analysis results from Tyser et al. and Zhang et al. are also consistent with classical fate mapping experiments in chick model (Stalsbert and Haan, 1969, summarised in Moorman et al. 2007, Figure 1,) demonstrating that the most cranial aspect of the heart-forming region (corresponding to the JCF) contribute to the sinus venous/ AVC and LV while more posterior regions contribute instead to the atria.

Thus, the Tyser et al and Zhang et al analysis (tracing the early mesoderm) together with our late *T* lineage tracing (tracing late mesoderm) are consistent with a model whereby the first progenitor to emigrate from the proximal PS are those contributing to the LV and AVC (populating anterior regions of the FHF) followed by cells contributing to the atria (summarised in figure 11C). In conclusion, we agree that our data cannot definitively exclude that earlier atrial progenitors exist. However, if these progenitors exist, they will represent a very minor proportion of the atria because most of the atrium is labelled when tamoxifen is administered at a late E7+7h timepoint (as we have already mentioned above, Figure 1 R1). We have discussed these important points in the Discussion.

*5) Following on from the above, a more detailed analysis needs to be conducted on the location of recombined/labelled cells. Given recombination takes place after 2 hours, analysis c/should be conducted 12 hours following tamoxifen administration in order to localize the cells.*

We thank the reviewer for these comments. We have included some of these experiments in Fig 5A-B, S7 A, C and D (for the *Foxa2* lineage trace) and Fig6A-C (for the *T*-lineage trace).

*This also needs to be conducted for the entire litter to obtain a convincing insight into what is being labelled.*

We do not think that imaging an entire litter will add much to the study since it will only show tdtomato positive cells populating the whole mesoderm (since *T* is a pan-mesodermal marker). Since *Foxa2* expression is restricted to the anterior primitive streak it was important to analyse the location of mesoderm descendants from the *Foxa2* expressing cells (Fig6A-B and S8B-C Fig). We have included an image below to illustrate this point:

*This is especially important given that the images of T-labelling seem to show it extending almost to the anterior of the embryo (Figure 3c) and it looks like T is strongly expressed in the mesodermal wing as supported by the scRNA-Seq atlas datasets referenced (Pijuan-Sala 2019) which show that T is most strongly expressed in mesoderm subtypes and not the PS between E7 and E7.75.  Detailed imaging, like that carried out for Foxa2, would allow the location of recombined cells to be identified and when combined with a mesoderm marker would further provide clarification as to whether these cells reside in the PS or are differentiating mesoderm.*

We apologise for the confusion. The embryos shown in Fig 4 have been tilted with a posterior orientation to show the entire width of the PS (similarly to Fig 5). The most anterior region of the embryo (*T* negative) is hidden. The location of *T* at EB-OB stages can be visualised in 3-dimensions here: <https://www.youtube.com/watch?v=CzRbIjphqEs&feature=emb_title> and in many more immunostaining and ISH from many other authors (for example: Arnold *et al.* 2009). We have added single optical sections in Fig S6. In addition, we have added some labelling to the Fig 3 to clarify the orientation of the embryos. The text for Fig 3 legend says: “Views are lateral/slightly posterior to show the whole width of the primitive streak”. *T* expression is an immediate early-response to mesoderm induction. *T* is expressed in the primitive streak and is retained when mesodermal cells exit the primitive streak in the nascent mesoderm (Fig 9B and C). Thus, our result show LV, RV, OFT/Atrial progenitors can be distinguished based on the timing of tamoxifen administration and ingress sequentially through the *T* positive primitive streak.

*6) Foxa2-labelling experiments: tamoxifen at E6+21 (Figure 4) resulted in ventricle and outflow labeling, however, the authors conclude Foxa2 is not expressed in the PS when the switch from ventricle to outflow/atria contribution occurs.  Given they detect both RV and outflow labelling, this suggests that outflow and ventricle are labelled early and specified later(?).*

We are unsure what the reviewer means here. We show that the OFT progenitors do not express Foxa2 when leaving the PS but are derived from a Foxa2 lineage (Fig 3C and Fig 6A). And we show that at least a subset of the RV progenitors must express *Foxa2* at the mid to late streak stage since they are recombining when tamoxifen is administered at E6+21h (Fig 3C).

*7) Based on the time course of tamoxifen administration the authors make the statement that atrial precursors are the last cardiomyocyte precursors to leave the PS. This is not strongly supported by the data given at E6+21 they observe atrial labeling, ie just because they are amongst the last progenitors to be restricted does not necessarily mean atrial precursors leave the streak and are specified earlier.*

We are unsure what the reviewer means here. Since we observed that atrial progenitors were the last to express *T*, we deduced that they were the last to leave the primitive streak (Fig 1C, D and E).

*Given clonal experiments have previously shown contributions to parts of the atria and LV are FHF-derived, how do the results herein fit with these prior studies?*

We thank the reviewer for this comment. We agree that this is an important point and we have now considered in the Discussion the left ventricle-atrial clones described in Devine *et al.* (see above in response to reviewer 2 and in our discussion).

*8) In a number of places important experimental details are lacking:*

*i) Whilst the text states 3494 single cell transcriptomes were obtained (page 14), what is the specific number used in this manuscript? i.e. how many FHF, pAHF, AHF cells were included, it appears less than 3494 in S4Fig. 4A, B?*

We have now provided the numbers on Fig 8A-B and in S8_source data.

*ii) Not enough details are provided in the text or legend to interpret Figure 5C and the tdTomato contributions to specific clusters.*

Thank you, we have now indicated the tdtomato contribution to specific clusters with more clarity in Fig 8D and E, S12C Fig, and S13A Fig.

*iii) The single cell RNA-seq analysis requires extended methodology- were the entire embryos dissociated?*

Yes.

*How many 10x runs were completed per group? Were each group included on the same 10x run?  Were there any batch effects and/or differences between groups?*

E6+21h, E7+3h datasets were performed once. Each group were included in the same sequence run. E7+14h was performed twice. E7+14h runs were collated using the Cell Ranger software as they were the same sample sequenced twice.

*What was the rationale for further sub clustering cells as stated in the text?*

We subclustered the data to better distinguish tdtomato positive from tdtomato negative subpopulations. The results support the conclusion, deduced form the lineage tracing experiments, that ventricular and outflow/atria progenitors are temporally segregated.

*Single cell analysis was done with a labelling regime not previously described (Collected at E6+21 but given tamoxifen at E6+5 (16 hours of labelling) and then collected at E7+3 but given at E7+3). How do these labelling regimes fit with the above lineage analysis conducted at different times?*

The important parameter here is the embryonic stage (OB-EB stages) rather than the time of collecting of the embryo (E7+3h in this case).

*iv) Detailed and specific details should be provided about the transcriptional state of identified precursor types - currently the data is not clear?*

*How many of each cell type are identified?*

We have provided the number of each cell type identified in S7_source data and S13A Fig.

*What is the complete unbiased list of genes which mark these clusters? Which genes overlap, and which do not?*

We have provided the unbiased list of genes which mark each cluster in S7_source data.

*9) Several statements are not supported by the data and especially given the relatively low number of embryos/hearts studied. For example,*

*i) Lines 200-202: " In 3/14 hearts, smaller tdtomato-expressing domains formed either in the left or right ventricular myocardium. This indicates that independent groups of Foxa2 expressing progenitors exist in the primitive streak that contribute to the left and right myocardium". This cannot be concluded on the basis of 3 hearts. especially if the labelled domains are small. This indicates that labelling might have been clonal or sparse, in which case it is likely to manifest in one or the other (but not both) ventricles, by chance, rather than because of different progenitors for LV and RV.*

We agree the labelling is sparse, but this was intended. The sparse labelling is likely to be clonal and provides evidence that independent progenitors for the LV and RV exist. We do not rule out the possibility of a common progenitor between LV and RV.

*ii) In several places the authors make inferences on dynamic behaviour eg "...forming a stream of migratory cells originating…", however this is on the basis of fixed samples and as stated above to make such claims the authors require time-lapse data on cultured embryos/hearts.*

We have now removed this statement (referring to a stream of migratory cells).

*Iii) Lines 291-294 : "We have summarised these results as a fate map for the different cardiac regions (Fig 7A-B) and we propose that right ventricle, outflow and atrial progenitors form molecularly distinct populations within the AHF and pSHF mirroring their distinct origin in the primitive streak." This claim is an over-interpretation; by definition, a fate mapping experiment is prospective. Much of the inferences in the accompanying figure are predicated on RNAseq and as such the authors have conflated the 'state' of a cell with its 'fate'. Again, this comes back to the main point above around validating the RNAseq data as relates to labelling of subpopulations in the PS, more refined lineage tracing and genetic targeting.*

We respectively disagree with the reviewer. Our data provide evidence that link the molecular and spatial origin of cells with their molecular and spatial fates. We contend that the Fig 11 A, B accurately summarises these results.

*10) The model in Figure 7 is surprisingly detailed given that there is no direct evidence for much of it in the preceding figures. The model shows relative positions of transcriptional sub-types, but there is no direct experimental evidence looking at their location. it also shows arrows indicating movement of cells, and again, there are no data presented to support the cell movements proposed.  The suggested validation of the sub-populations within the PS (point 1) above) to include more refined lineage tracing, arguably also requires time-lapse experiments on cultured embryos/ hearts to capture patterns of migration and contributions to the forming heart to support the model proposed.*

Thank you for this suggestion. We have now included a time lapse movie in the manuscript (Fig 10). The migration of the mesodermal cells is consistent with those we deduced from our *T* and *Foxa2* lineage tracing and are also consistent with previous fate mapping results (Kinder et al. 2002 Trends Cardiovasc Med 2001;11:177–184 Figure 3).

*Minor points:*

*-	Brightfield images of embryos in Figure 1H are not very clear and do not adequately allow staging to be assessed. Given the use of the T-GFP reporter, images of T expression would also be helpful here.*

Unfortunately, we did not take GFP images of these embryos.

*-	Additional clarification around the precise staging is required: what does a late streak or mid-streak embryo refer to, e.g. E6+21, E7, E7+7, E7+11?*

This is described in Fig 1G and H.

*How do distal and proximal PS relate to anterior PS and timeframes when cells enter the streak?*

Distal and proximal refer to the embryonic axis. The anterior PS refers to a subpopulation of primitive steak cells at the MS-LS stages located distally and marked by the expression of *Foxa2* (in addition to other genes, see Fig 9B).

*-	Figure 4A - the masked image is confusing and it looks like only the td-tomato image is masked, why?*

We masked the tdtomato+/Foxa2+ cells so that the relevant data are clearer to the reader.

*Panel iii is not convincing given there appear to be penetration issues as DAPI is not present, what about the GFP signal?*

We do not understand this point. The GFP signal is now shown in the new Fig6. We did not use DAPI in this experiment since all four channels were already taken.

*-	Figure 5B shows E7+14 embryos but this is not discussed in the text or legend.*

The E7+14h embryos shown in the new Fig 8A are mentioned in the legend and in the text.
