# Supplementary material for: Ventricular, atrial, and outflow tract heart progenitors arise from spatially and molecularly distinct regions of the primitive streak
Source: PLoS Biol. 2021 May 17;19(5):e3001200. doi: 10.1371/journal.pbio.3001200 (PMC8158918; doi:10.1371/journal.pbio.3001200)
Supplement: Attachment — Submitted filename: minor revisions R3.docx [file pbio.3001200.s027.docx]

Dear Editorial Team,

Thank you for the editorial decision. We are very pleased. Please find below the point-by-point rebuttal for the minor revisions.

Best wishes,

Kenzo Ivanovitch

Thank you for providing the individual numerical values that underlie the summary data displayed in the figures to comply with our data policy. Nevertheless, we are missing the data of some of the figure panels and we would like you to clarify/address a few points:

Fig. 9E, G, H; Fig. S12B, D; Fig. 13B and Fig. 15A, B

We apologise for these omissions.

The table of gene expression for the heat map shown in Fig. 9E is now in S9_source data.

Values for the violin plots shown in Fig9 G-H and S15A, B Fig are now in S9_source data.

Mean expression and percentages of expressing cells for the dot plots in S12D Fig and S13B Fig are now also in S9_source data.

S12B Fig are feature plots and the reads counts per gene per cell is accessible in NCBI under the accession number GSE153789.

- Some of the columns in Fig. 5E and 5F have exactly the same values, but are labelled differently. Are they supposed to be the same (if so, please explain) or is this a mistake?

Yes, this is correct. Fig5E and Fig5F are different analyses of the same dataset.

Fig. 5E depicts Foxa2 and T intensity relative to the position of the cell (for z=10).

Fig 5F depicts T intensity relative to Foxa2 intensity for tdTomato+, Distal PS and Proximal PS cells (for z=10, 12 and 16).

- In S9 source data, there are two tabs named Fig. 6B and Fig. 6C, but they seem to belong to Fig. 9B and C – is this correct? If so, please use the correct labels for the tabs and the data titles. Otherwise, please add the missing data for Fig. 9C.

This is correct. We apologise for the mistake. We have now corrected the labels.

- Please also ensure that figure legends in your manuscript include information on WHERE THE UNDERLYING DATA CAN BE FOUND.

This has now been done.

- In your Data Availability Statement you mention that the single cell RNA sequencing data have been deposited in NCBI under the accession number GSE153789. Please make sure that this data is made publicly available before the manuscript is accepted for production.

We confirm the data are now publicly available under the accession number GSE15378.
